# Public patient forwarding to private pharmacies: an analysis of data linking patients, facilities and pharmacies in the state of Odisha, India

**DOI:** 10.1136/bmjgh-2024-017788

**Published:** 2025-02-18

**Authors:** Annie Haakenstad, Anuska Kalita, Bijetri Bose, Arpita Chakraborty, Kirti Gupta, Sian Hsiang-Te Tsuei, Liana Rosenkrantz Woskie, Winnie Yip

**Affiliations:** 1 Institute for Health Metrics and Evaluation, University of Washington, Seattle, Washington, USA; 2 Global Health and Population, Harvard T H Chan School of Public Health, Boston, Massachusetts, USA; 3 University of California Los Angeles Jonathan and Karin Fielding School of Public Health, Los Angeles, California, USA; 4 Oxford Policy Management, 4/6 First Floor, Siri Fort Institutional Area, New Delhi, India; 5 The University of British Columbia Department of Family Practice, Vancouver, British Columbia, Canada; 6 Simon Fraser University Faculty of Health Sciences, Burnaby, British Columbia, Canada; 7 Department of Community Health, Tufts University, Boston, Massachusetts, USA

**Keywords:** India, Health policy, Health services research, Cross-sectional survey, Health systems

## Abstract

**Introduction:**

In India, public sector patients purchase drugs from private pharmacies instead of obtaining them for free from public pharmacies—a phenomenon we call public patient forwarding to private pharmacies. This behaviour results in substantial financial hardship. We examine whether low public drug stocks, patient preferences for private drugs or the presence of private pharmacies nearby explain this behaviour.

**Methods:**

We collected cross-sectional data from 7567 households, 523 health facilities and 1036 private pharmacies in Odisha, India. We linked 917 outpatient visits to facilities based on patient reports and linked public facilities to the nearest private pharmacy using Global Positioning System coordinates. We used ordinary least squares regression to assess whether the behaviour of facilities and patients was associated with drug stocks and pharmacy proximity, and whether patient satisfaction was associated with private drug purchases.

**Results:**

Among public patients prescribed drugs, more than 70% purchased private drugs. In hospitals, for each 10% increase in drug stocks, 4.8% fewer patients purchased private drugs (p=0.047). In primary facilities, the same share of patients purchased private drugs across stock levels. Regardless of facility level, when more than 75% of drugs were in stock, 60% or more of patients still obtained drugs from the private sector. Patients were more likely to purchase private drugs when private pharmacies were near public facilities, but were not more satisfied with their visit when they obtained private drugs.

**Conclusion:**

The results suggest that private pharmacies are both secondary and complementary suppliers of drugs for hospitals, but may act more like substitutes for primary facilities, consistent with evidence that private pharmacies provide advice and other services akin to primary care in Odisha. Improving public facility drug stocks alone is unlikely to fully address drug-driven financial hardship in India. Provider prescribing practices should be investigated to identify additional policy options.

**WHAT IS ALREADY KNOWN ON THIS TOPIC**

- Public patient purchasing of drugs from private retail pharmacies is a common phenomenon in India and other countries that contributes substantially to financial hardship.
- While a number of commentaries and theoretical pieces have hypothesised why this occurs, no existing studies empirically examine drivers of this behaviour.

**WHAT THIS STUDY ADDS**

- This study deploys a novel data set linking patients, facilities and private pharmacies to assess whether drug stocks, patient preferences and the proximity of private pharmacies are associated with private drug purchases among users of public outpatient care.

**HOW THIS STUDY MIGHT AFFECT RESEARCH, PRACTICE OR POLICY**

- Improving public drug stocks will reduce some but far from all private drug purchases among public patients.
- Other policies, such as examination of public drug lists, insurance coverage of private drugs and better enforcement of generic prescription mandates, should be considered to address financial hardship due to healthcare costs in India.

## Introduction

 Drug spending is a key cause of out-of-pocket (OOP) expenditure around the world, threatening financial protection and the pursuit of universal health coverage.^1^ High drug spending is a particularly large problem in India, where spending on pharmaceuticals comprises nearly 70% of all OOP costs,^2^ and 16% of households face catastrophic health expenditure (CHE) at the 10% level each year.^3^ Understanding why OOP spending on drugs is so high is crucial to improving health system performance globally and in India.

Large OOP drug spending in India is puzzling for several reasons. First, India is one of the largest producers by volume of pharmaceuticals in the world,^4^ the majority of which is consumed in the domestic market.^5^ Second, the Indian government imposes price controls on hundreds of essential medicines, which has lowered the price of drugs at point of purchase to among the lowest in the world.^6 7^

Beyond prices, government policy in many Indian states is to provide drugs for free in public facilities,^8 9^ which is true in our study setting, the state of Odisha.^10^ This, however, is a policy that most Indian consumers do not or cannot take advantage of. Among patients using the public sector for outpatient care, a substantial share purchase drugs in the private sector^11^—a phenomenon we refer to as public patient forwarding to private pharmacies. Eliminating spending on private retail drugs could reduce CHE rates by more than half in Odisha.^11^

Existing studies offer theories as to why public patient forwarding occurs, which we summarise and expand on in the next section. However, to date, no studies have empirically examined its causes in India, primarily because few data sources capture where patients obtain drugs. In a 2022 review of 123 studies on CHE in India, we found information on where patients obtain drugs in just two peer-reviewed articles.^11^ These studies assessed this behaviour only in a single cell in a table or for one disease area only and included no discussion of the behaviour or its drivers.^12 13^ An evaluation study of shops providing discounted drugs also noted where populations obtain drugs,^14^ but, again, the discussion of public patient forwarding was limited.

Thus, in this study, we investigate one of the key underlying drivers of financial hardship in India by assessing public patient forwarding. We use a unique data set linking patients, public facilities and private retail pharmacies collected over 2019/2020 in the Indian state of Odisha. Our main measure of public patient forwarding is whether patients who have an outpatient consultation with a public provider obtain drugs from a private retail pharmacy. Using ordinary least squares (OLS) regression, we examine whether public patient forwarding is more likely when fewer drugs are in stock at facilities. We also test whether patient satisfaction ratings are consistent with patients being happier when they purchase private drugs. Finally, we test whether these effects are amplified when private pharmacies are nearby. We conclude by discussing how the results could be used to improve financial risk protection in India.

## Hypotheses

### Low drug stocks

A key explanation for public patient forwarding is that public sector drug stocks are not available to meet patient needs. Patients must therefore obtain drugs elsewhere, with the main alternative being private retail pharmacies.^9^ Such an explanation conceives of patients first ‘shopping’ at the public pharmacy and, if they cannot obtain the drugs they need there, they resort to the private pharmacy as a second choice.^15^ This sequence of behaviour makes sense when: (1) we believe patients are price-sensitive; and (2) drugs are free or highly subsidised in the public sector versus full price in the private sector,^16^ which is the case in our study setting. From such a perspective, private retail drug stocks serve as a backstop, backfilling the public drug supply.

### Private drug preference

The ‘backfill’ explanation ignores the role of patient preferences for private drugs however.^17^ If patients prefer private drugs over public drugs, they may bypass public pharmacies altogether, proceeding directly to private pharmacies without first ‘shopping’ at the public pharmacy. Indeed, there is evidence that Indian consumers strongly prefer private drugs over public drugs and they are not perfect substitutes: public pharmacies tend to stock generic drugs while private retail pharmacies tend to stock branded and branded generic drugs.^9 18^ Despite evidence that branded drugs and generic drugs have the same chemical composition,^19^ and no difference in adverse side effects,^20^ providers and patients report perceiving branded and branded generic drugs to be of higher quality and more effective than generic or so-called ‘public’ drugs.^17 21^ Private drug purchases could thus be consistent with patients maximising the (perceived) health benefits of medicine.

Patient perceptions of quality by facility level may also drive differences in behaviour. The frequent bypassing of primary facilities in India suggests that patients believe hospitals provide better care than primary facilities.^22^ Should these beliefs extend to patient perceptions of the drug supply, patients may believe that hospital drugs are better than primary facility drugs. This could lead patients to do more ‘shopping’ at hospitals and more bypassing of public pharmacies in primary facilities.

### Private pharmacy convenience as an amplifier

A secondary set of hypotheses concerns the practice of private pharmacies locating themselves just outside public facilities. The proximity of private pharmacies to public facilities could change the behaviour of patients. Patients may be more likely to translate their preference for private drugs into the action of bypassing a public pharmacy when there is little cost to visiting a private retail pharmacy, for example, if it is conveniently located nearby.

The proximity of private pharmacies to public facilities may also be linked to low public drug stocks.^23^ Private pharmacies may choose to locate near public facilities that have difficulty keeping stocks high to secure a place as the second choice for a steady stream of customers. Alternatively, private retail pharmacies may induce demand for private drugs in situating themselves near public facilities by changing the behaviour of public facility staff. When private drugs are easy for patients to obtain, public facility providers prioritising tasks other than stocking and dispensing drugs (eg, treating patients) could be a rational behaviour that maximises social welfare.^15 24^ In small public facilities with few personnel, staff may have to trade off time between drug dispensing and stocking tasks versus consulting with patients.

Finally, private retail pharmacies could influence the behaviour of facility staff by compensating them when patients purchase private drugs,^25^ and the effect could be stronger when private pharmacies are nearby. Private pharmacies may offer a commission for referrals that result in drug sales or partial ownership of pharmacies so that that providers have an incentive to generate sales for private pharmacies.^26^ Public facility staff might thus purposefully keep public stocks low, creating conditions that make it likely that patients use the nearby private pharmacy. Public facility providers may also directly refer patients to particular pharmacies.^27^ While compensation could occur regardless of the distance to the nearest pharmacy, nearby private pharmacies may be better able to capture demand from indirect or direct referrals.

## Materials and methods

### Data

Over 2019–2020, we collected data from 7567 households, 523 government-run health facilities and 1036 retail pharmacies in Odisha. The method of sampling and mode of data collection have been described elsewhere.^11^ We summarise our methods here and provide additional details in the online supplemental appendix.

Households were selected based on multistage, stratified, probability proportionate to size sampling, with oversampling of households who had chronic conditions or any encounter with the health system. Survey weights were developed based on iterative proportional fitting to ensure results are state representative. The household survey collected information on health system encounters for every individual in the household. Rupees were converted to US dollars using the 2019 average exchange rate of US$0.014 to rupee.^28^

We sampled public health facilities according to their designated level. In the districts sampled for the household survey, we surveyed all hospitals and community health centres (CHCs), which we group together and refer to as hospitals in this study. All primary facilities (comprised of primary health centres, health and wellness centres and subcentres) in the blocks where households were surveyed were included in the study (more information in section C of the online supplemental appendix). We created a sampling frame for private pharmacies using several methods. We asked household survey respondents which private pharmacies they used; we also mapped and sampled all private retail pharmacies within 3 km of each public facility surveyed and identified additional pharmacies using snowball methods. We sampled up to 10 private pharmacies. Surveyors interviewed facility or pharmacy staff and directly observed whether essential medicines were in stock in the facility on the day of the survey, with a different list of drugs used for hospitals than primary facilities (full list in the online supplemental appendix).

We linked 917 outpatient visits to 143 public facilities using the facility name, address and facility location reported by patients. This includes 654 patients in 55 hospitals and 263 patients in 88 primary facilities. We calculated the distance between Global Positioning System coordinates of facilities and private retail pharmacies using the geodist function in Stata.^29^

### Analysis

First, we tested whether private drug purchases among public sector patients are sensitive to drug stocks, controlling for the distance between public facilities and private pharmacies. To do so, we used the sample linking facilities, patients and private pharmacies. If private drug purchases are more likely to take place when fewer drugs are in stock, this would represent private pharmacies backfilling the public drug supply. If more private drug purchases occur where private pharmacies are nearby, this would suggest the proximity of private pharmacies is amplifying public patient forwarding. We note that both hypotheses could hold simultaneously, given the potential for private pharmacies to induce low drug stocks. In secondary analyses, we examined CHE due to drugs, referrals to private pharmacies and the number of drugs obtained, to assess patient welfare.

We employed OLS regressions and controlled for individual, household and facility characteristics that were statistically significantly different in the full versus linked samples, fixed effects on facility level, as well as variables capturing pharmacy management and supply chains: electronic ordering of drugs, computerised tracking of drugs, receipt of drugs takes 2 weeks or more and whether the closest pharmacy was a Jan Aushadhi pharmacy. To test the robustness of our results, we also fit logistic, generalised estimating equation (GEE) and generalised linear binomial models (GLM) with data collapsed by facility (results reported in the online supplemental appendix). Wherever we used individual-level data, we clustered SEs by facility and used survey weights that make our sample nationally-representative.

Second, we analysed patient satisfaction following a public sector outpatient visit. Should patients have a private drug preference, we would expect them to be equally or more satisfied with their public healthcare encounter when they obtain private drugs as compared with when they obtain public drugs only. Patients reported their satisfaction with their visit according to a four-point scale. We created an indicator variable for patients selecting ‘excellent’ or ‘good’ in response to: (1) ‘Overall, thinking about your visit, please rate how well the care you received met your health needs. That is, how much did the visit help solve your health problem or help you feel better?’ and (2) ‘Overall, taking everything into account about your visit, how would you rate the quality of care you received?’ Our main predictors of interest were whether any drugs or any private drugs were obtained. We used an OLS regression, controlling for OOP spending, whether any tests were obtained as well as individual and household-level characteristics. We tested the robustness of our results with logistic, GEE and GLM methods (results in the online supplemental appendix).

Third, we considered whether fewer public drugs were in stock when a private pharmacy was nearby—this would be consistent with private pharmacies choosing to locate near public facilities with low drug supply as well as private pharmacies inducing public staff to neglect drug stocks. We used the facility-pharmacy linked sample to regress the share of essential medicines in stock on the natural-log transformed distance to the closest pharmacy with a 10 m offset in an OLS regression. Our analysis was stratified by level of facility (hospitals, primary facilities) and included facility-level fixed effects for facility level. We control for all facility characteristics that were statistically significantly different between the linked and full samples and pharmacy management covariates, as above.

We report regression results using three sets of controls: (1) basic, (2) sample difference and (3) extended and/or pharmacy management controls, with each additional set including the previous set of controls (so the regression using sample difference controls also includes basic controls). This reporting approach enables us to represent the extent to which our results are sensitive to the characteristics of individuals and facilities. Unless otherwise noted in the text, we report the results for the most extensive set of controls in the results section. All analyses were conducted in Stata (V.15.1).

## Results

Across facilities, nearly 90% of public outpatient users obtained any drugs (an average of three drugs per person), with more than 70% of those patients purchasing private drugs (online supplemental appendix table G3). More than 10% of outpatient users faced CHE due to drugs. Almost 15% of patients were referred to a particular pharmacy. Primary facilities were much more likely to be in rural areas as compared with hospitals (98% vs 68%) (online supplemental appendix table G4). The full and linked patient and facility samples were similar across most dimensions, and the appendix discusses the relevant dissimilarities (online supplemental appendix page 22).

**Figure 1:**
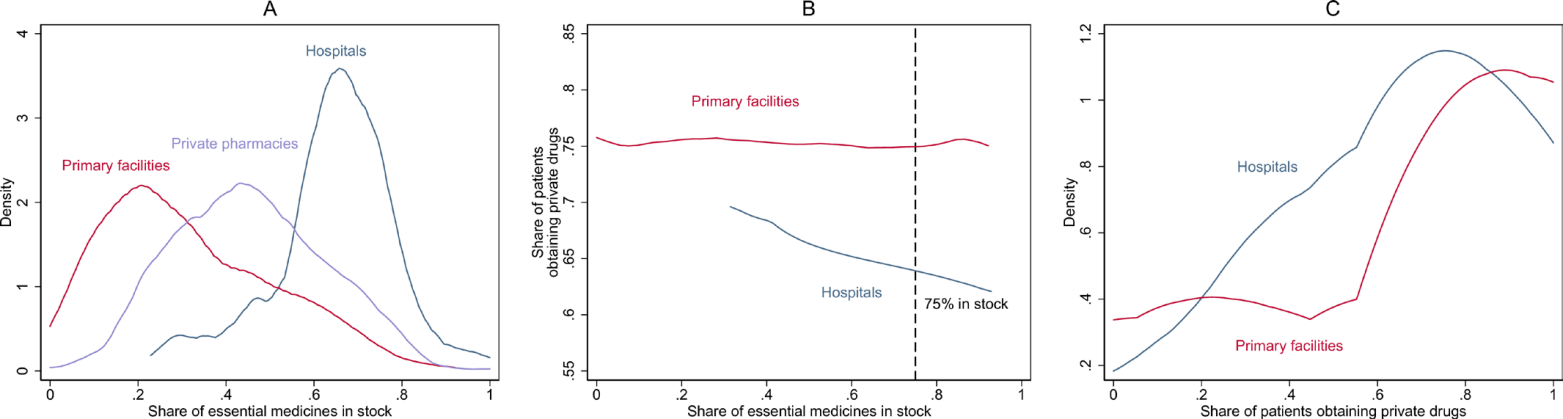
Distribution of the share of essential medicines in stock (**A**), private drug purchases versus share of essential medicines in stock (**B**) and distribution of private drug purchases (**C**), by level of facility. Notes: hospitals (blue) include hospitals and community health centres. Primary facilities (red) include primary health centres, health and wellness centres and subcentres. List of essential medicines directly observed as present on the day of the survey listed in the appendix. The list of drugs assessed is different for public primary (41) versus public hospitals and community health centres and private pharmacies (58) because not all medicines stocked by the tertiary facilities are expected to be stocked by the primary level facilities. Epanechnikov kernel density was used for A with bandwidth based on minimising the mean integrated square error. Local polynomial with bandwidth of 0.2 used for B. Epanechnikov kernel density with bandwidth of 0.2 used for C. Panels A and C based on data collapsed by facility, whereas panel B uses patient-level data.

Drug supply chain operations were distinct in public facilities versus private pharmacies (online supplemental appendix table G4), which could have a bearing on drug stocks. Computerised tracking of drug stocks was common in public hospitals (72%), but not in private retail pharmacies (18%) or public primary facilities (4%). Hospitals had more drugs in stock (64%) than private retail pharmacies (48%), with primary public facilities having fewer still (32%) (figure 1A).

Low drug stocks: our regression analysis provides some evidence of private pharmacies backfilling for hospitals. Fewer patients in hospitals obtained private drugs as the share of medicines in stock increased (figure 1B): a 10 percentage point increase in drugs in stocks in hospitals was associated with an estimated 4.8 percentage point decline in private drug purchases (table 1, p=0.047) when we include sample difference controls, although this result is not significant when including pharmacy management covariates (extensive controls). A 10% point increase in hospital drugs stocks was associated with a 3.7 percentage point reduction in CHE (p=0.033) when only basic controls are used, with a non-significant result when extensive controls are included (table 1).

**Table 1:** Coefficient estimates from ordinary least squares regression of outcomes on drug stocks and distance to nearest pharmacy, with controls, by level of facility

	(1)	(2)	(3)	(4)	(5)	(6)	(7)	(8)	(9)	(10)	(11)	(12)
	**Hospitals**											
	Private drugs purchased			CHE due to drugs			Number of drugs			Referred to a pharmacy		
Share of drugs in stock	−0.452*	−0.481*	−0.396	−0.373*	−0.327	−0.405	−1.223	−0.950	−0.788	−0.349	−0.313	−0.191
	(0.198)	(0.236)	(0.255)	(0.170)	(0.206)	(0.222)	(0.890)	(1.043)	(0.996)	(0.247)	(0.281)	(0.293)
Log distance to nearest pharmacy (km)	−1.896*	−1.598	−1.696*	0.0994	0.108	0.107	−2.607**	−2.583***	−2.525***	−0.855	−0.717	0.0460
	(0.822)	(0.891)	(0.709)	(0.135)	(0.116)	(0.118)	(0.843)	(0.691)	(0.511)	(0.481)	(0.500)	(0.536)
N	569	567	567	598	596	596	597	595	595	569	567	567
	**Primary facilities**											
	Private drugs purchased			CHE due to drugs			Number of drugs			Referred to a pharmacy		
Share of drugs in stock	0.233	0.330	0.366	0.199	0.176	0.0866	1.232	1.055	1.034	−0.381*	−0.508*	−0.280
	(0.157)	(0.196)	(0.268)	(0.109)	(0.128)	(0.163)	(0.830)	(0.796)	(1.007)	(0.172)	(0.208)	(0.232)
Log distance to nearest pharmacy (km)	−0.104*	−0.109*	−0.0190	−0.066**	−0.089***	−0.081**	−0.0703	−0.036	−0.023	−0.036	0.0088	0.023
	(0.043)	(0.053)	(0.048)	(0.021)	(0.024)	(0.030)	(0.115)	(0.115)	(0.131)	(0.032)	(0.037)	(0.051)
N	234	228	208	245	239	219	245	239	219	234	228	208
Basic controls	X	X	X	X	X	X	X	X	X	X	X	X
Sample difference controls		X	X		X	X		X	X		X	X
Extended and pharmacy management controls			X			X			X			X

Notes: Standard errorStandard error in parentheses. CHE: catastrophic health expenditure measured at the 10% of consumption expenditure threshold level. Regression coefficients and standard errorsSEs estimated from an ordinary least squares regression using survey weights and robust standard errorsSEs clustered by facility. All regressions included facility -level fixed effects, illiteracy and natural log -transformed non-health expenditure per household member. Sample difference covariates included all indicators that were statistically significantly different between the all-facility sample as compared tocompared with the sample which included linked patients and facilities only. These covariates for hospitals were: gender, poor/fair self-rated health, diagnosis with a chronic condition, closest government facility used, use of care for fever or childbirth, whether the facility had an inpatient department. In primary facilities, sample difference covariates included: rural residence of patient, the natural log of the number of staff present on the day of the survey and whether the facility faces severe water shortages. Extended covariates included sample difference covariates identified in both levels and pharmacy management covariates: electronic ordering of drugs, computerizedcomputerised tracking of drugs, receipt of drugs often takes more than two2 weeks and whether the closest pharmacy was a Jan Aushadhi pharmacy. P- values: * p*p<0.05, ** p**p<0.01, ***p***p<0.001.

However, two pieces of evidence suggested that drivers other than backfilling are operating. First, among primary facility patients, no association was detected between the proportion of drugs in stock and whether private drugs were obtained (figure 1B, table 1). Second, although the share of patients obtaining private drugs varied widely by facility (figure 1C), more than 60% of the patients still sought drugs from the private sector even when hospitals and primary facilities had more than 75% of drugs in stock (figure 1B).

Private drug preference: our analysis provides no evidence that patients are more satisfied when they obtain private drugs (table 2). Obtaining drugs from the private sector was associated with lower ratings of met needs among patients of primary care facilities (−0.190, p=0.029). The negative association is robust to restricting to primary patients who selected a facility with a convenient private pharmacy (within 500 m) (table 2), who we might expect to have a stronger preference for private drugs.

**Table 2:** Coefficient estimates from ordinary least squares regressions of patient ratings on private drugs obtained and any drugs obtained, with controls, by level of facility

	(1)	(2)	(3)	(4)	(5)	(6)	(7)	(8)
	**Hospitals**							
	Rating of excellent or good needs met				Rating of excellent or good overall quality			
Private drugs obtained	−0.016	−0.020	−0.039	−0.023	0.015	0.011	−0.022	−0.009
	(0.041)	(0.040)	(0.051)	(.053)	(0.049)	(0.049)	(0.058)	(0.059)
Any drugs obtained	0.217**	0.218**	0.268**	0.273**	0.189*	0.184*	0.288**	0.296**
	(0.077)	(0.075)	(0.096)	(0.095)	(0.084)	(0.082)	(0.096)	(0.097)
N	900	898	552	552	900	898	552	552
	**Primary facilities**							
	Rating of excellent or good needs met				Rating of excellent or good overall quality			
Private drugs obtained	−0.124*	−0.132*	−0.190*	−0.244*	−0.033	−0.066	−0.066	−0.0412
	(0.053)	(0.055)	(0.086)	(0.112)	(0.062)	(0.062)	(0.107)	(0.144)
Any drugs obtained	−0.017	−0.001	0.129	0.011	0.0162	0.031	0.112	−0.113
	(0.058)	(0.058)	(0.112)	(0.144)	(0.108)	(0.100)	(0.149)	(0.212)
N	430	430	219	125	430	430	219	125
Basic controls	X	X	X	X	X	X	X	X
Sample difference + extended controls		X	X			X	X	
Facility characteristic controls			X				X	
Pharmacy <500 m				X				X

Notes: Standard errorStandard error (SE) in parentheses. Basic controls included: whether a test was obtained; facility fixed effects; log non-health consumption expenditure; log health spending per visit with a 10% of the median offset; and illiteracy status. Sample difference covariates included all indicators that were statistically significantly different between the all-facility sample as compared tocompared with the sample which included linked patients and facilities only. Sample difference controls included: whether the patient reported poor or fair health; sex; whether the patient reported belonging to the scheduled tribe or scheduled caste groups, respectively; rural location; whether care was sought at the closest government facility; whether care was sought for childbirth or fever (respectively); and whether the patient was diagnosed with a chronic condition. Facility characteristic controls were also included for the subset of patients that could be linked with a facility: log number of staff present on the day of the survey; whether the facility provides inpatient care and faces severe water shortage. Standard errorsSEs were clustered by primary sampling unit and linked facility. P values: *p<0.05, **p<0.01, ***p<0.001.

**Figure 2:**
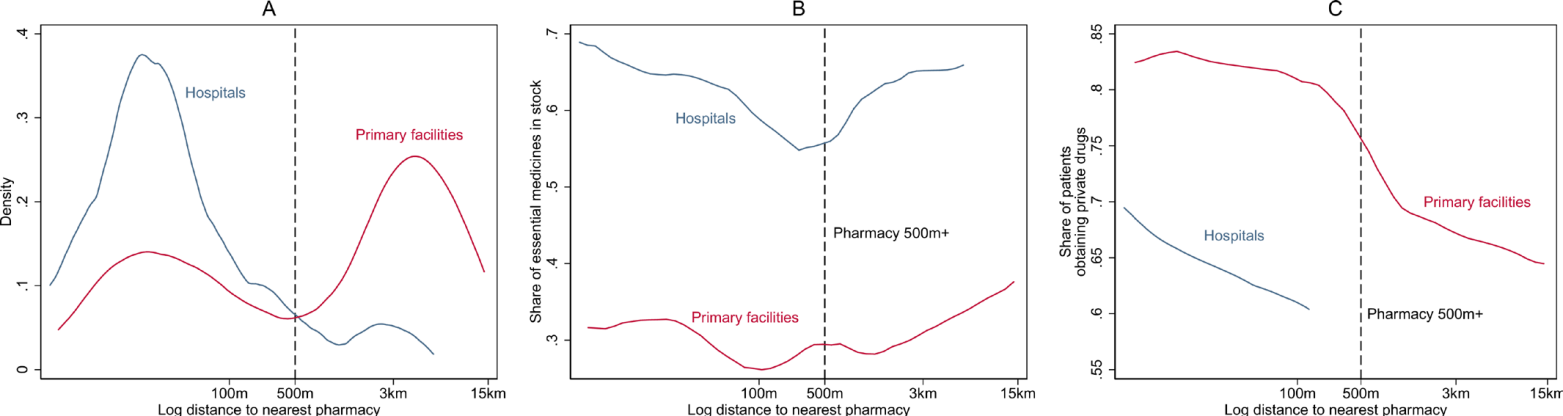
Distribution of distance to nearest private pharmacy (**A**), drug stocks versus distance to nearest private pharmacy (**B**) and private drug purchases versus distance to nearest private pharmacy (**C**), by level of facility. Notes: hospitals (blue) include hospitals and community health centres. Primary facilities (red) include primary health centres, health and wellness centres and subcentres. Distance between health facility and pharmacy was calculated using Global Positioning System coordinates and the geodist function in Stata. List of essential medicines directly observed as present on the day of the survey listed in the appendix. The list of drugs assessed is different for public primary (41) versus public hospitals and community health centres and private pharmacies (58) because not all medicines stocked by the tertiary facilities are expected to be stocked by the primary level facilities. Epanechnikov kernel density was used for A with bandwidth based on minimising the mean integrated square error. Local polynomial with a bandwidth of 0.5 (panel B) and 1 (panel C) used. Top 1% of distance measures were trimmed. An offset of 10 m was added to nearest distance to enable the inclusion of zeroes. Offset is taken into account in all distance labels. Panels A and B made with data collapsed by facility. Panel C was constructed with individual-level patient data.

Private pharmacy convenience as an amplifier: our analysis provides evidence of differences in facility characteristics and patient behaviour when a private pharmacy is nearby as compared with far away, although the level of facility was also important. First, 84% of hospitals had a private retail pharmacy within 500 m (IQR of 25–205 m) as compared with 58% of primary facilities (IQR of 69 m to 5.3 km), as shown in figure 2A. Drug stocks in hospitals changed little regardless of the distance to a private pharmacy (table 3, p=0.659). In contrast, primary facility stocks were higher when private pharmacies were farther away: an 8.2 percentage point increase in primary stocks was estimated for an additional kilometre in distance to the nearest private pharmacy relative to the 25th percentile of the distance of 69 m (table 3, p=0.002, figure 2B). This is a 26% increase over average primary facility drug stocks (32%).

**Table 3:** Coefficient estimates from ordinary least squares regression of share of essential medicines in stock, with controls, by level of facility

	(1)	(2)	(3)	(4)	(5)	(6)
	**Hospitals**			**Primary facilities**		
Log distance to nearest pharmacy (km)	−0.011	0.0068	0.014	0.018*	0.022*	0.030**
	(0.028)	(0.031)	(0.031)	(0.009)	(0.009)	(0.010)
Sample difference controls		X	X		X	X
Extended and pharmacy management controls			X			X
N	117	116	116	395	388	332

Notes: Standard errorStandard error (SE) in parentheses. Regression coefficients and standard errorsSEs estimated from an ordinary least squares regression with robust standard errorsSEs. Sample difference covariates included all indicators that were statistically significantly different between the all facility sample as compared tocompared with the sample which included linked patients and facilities only. These controls for hospitals were: gender, poor/fair self-rated health, diagnosis with a chronic condition, closest government facility used, use of care for fever or childbirth, whether the facility had an inpatient department. In primary facilities, sample difference covariates included: rural residence of patient, the natural log of the number of staff present on the day of the survey and whether the facility faces severe water shortages. Extended covariates included sample difference covariates identified in both levels and pharmacy management covariates: electronic ordering of drugs, computerizedcomputerised tracking of drugs, receipt of drugs often takes more than two2 weeks and whether the closest pharmacy was a Jan Aushadhi pharmacy. P- values: * p*p<0.05, ** p**p<0.01, ***p***p<0.001.

Fewer patients obtained private drugs when private pharmacies were farther away from hospitals and primary care facilities (figure 2C). As table 1 shows, an increase in distance to the nearest pharmacy was associated with a large reduction in purchasing any private drugs among hospital patients (p=0.032) and a more minor reduction among primary facility patients (p=0.041) with basic and sample difference controls only. Among primary facilities, the coefficient becomes insignificant when all controls are included, suggesting the pharmacy difference covariate, like the computerised tracking of stocks, may explain some of this association, but we note the large number of additional controls included (10) and the small sample size (208 patients) is also likely to play a role. Lower CHE was associated with increased distance to a private pharmacy among primary facility patients (−0.089, p<0.001) but not among hospital patients (0.108, p=0.354). Primary care patients did not obtain fewer drugs when private pharmacies were farther away, whereas hospital patients did (p<0.001). Overall, regardless of the distance to the nearest pharmacy, more than 60% of patients obtained private drugs (figure 2C).

We did not find evidence of direct compensation methods of providers by pharmacies, but we are limited in the ways we test for this behaviour. Few private retail pharmacies reported having an owner than worked in a public facility (1%) (online supplemental appendix table G4). We also did not find an association between distance to pharmacy and referrals (table 1). Public primary facility patients were more likely to be referred to a pharmacy when fewer drugs were in stock although this was not robust to the full set of controls (table 1).

## Discussion

Using a unique, linked data set that captured patient satisfaction and characteristics of private pharmacies and public facilities, we empirically tested several hypotheses explaining public patient forwarding.

Low drug stocks: a key question examined in this study was whether low public drug stocks drive patients to the private sector. We find that, in hospitals, where more drugs were in stock, patients were less likely to obtain private drugs. This indicates that at least some patients in hospitals do not directly bypass the public pharmacy at the hospital but rather ‘shop’ at the public pharmacy before resorting to the private sector.^16^ We do not find evidence of private drug purchases responding to drug stocks in primary facilities. These differences are consistent with the patient perceptions of higher quality of care in hospitals versus primary facilities extending to drug stocks,^22^ but there are other potential explanations discussed below. Overall, our analysis suggests that the ‘backfill’ explanation—that private pharmacies serve as a second choice, backstopping the public drug supply—applies for some hospital patients but is less likely for patients at primary facilities.

Private drug preference: we posited that patients bypass public pharmacies because they prefer private drugs over public drugs. We found that, in hospitals, patients did not report different satisfaction ratings when private drugs were purchased, consistent with this hypothesis. Primary facility patients, however, were less likely to feel their visit met their needs when they obtained private drugs. Primary facility patients may have weaker preferences for private drugs, perhaps because they are more budget-constrained or have different expectations for care as compared with hospital outpatient users.

Private pharmacy convenience as an amplifier: a secondary question tackled in this study was whether the proximity of private retail pharmacies to public facilities amplifies public patient forwarding. We find that, in both hospitals and primary facilities, more patients purchased private drugs when private pharmacies were nearby. In hospitals, increased distance to private pharmacies was also associated with fewer drugs purchased overall. Private pharmacies may act as both secondary and complementary suppliers to hospitals: a second choice for patients when drugs are out of stock (fulfilling a backfill role) but also a provider of some drugs that hospitals do not stock.

Overall, we are not able to pinpoint the underlying reasons that pharmacy proximity amplifies public patient forwarding. It is unclear whether this is due to private pharmacies’ influence on drug stocks, activation of patient preferences or other reasons.

First, we found that when private pharmacies were closer to primary facilities, public drug stocks were slightly lower. The substitution of private stocks for public stocks is consistent with evidence that private pharmacies provide advice and other services akin to primary care in Odisha.^18 23^ Our analysis cannot fully disentangle why—private pharmacies may select to locate near primary facilities that struggle with stocks or primary facility staff behaviour may be affected by pharmacy proximity.

In contrast, analysis of hospitals showed no association between the level of drug stocks and pharmacy proximity. Given the large volume of patients, pharmacies may select to locate near a hospital regardless of the hospitals’ stocking patterns. Hospitals may also be less likely to de-prioritise drug stocks due to pharmacy proximity. The presence of dedicated pharmacy staff at hospitals means there is no need to consider trade-offs between consultations and drug stocking activities as is likely the case in primary facilities.

In terms of other hypotheses, we did not find much support for the hypothesis that pharmacy proximity activates patient preferences for private drugs. Analysis of pharmacy referrals and pharmacy ownership also did not provide evidence that incentives from private pharmacies shaped provider behaviour, although we were limited to examining provider referrals to particular pharmacies and reports of ownership. Further investigation should be done into private pharmacy incentives.

Alternative explanations for public patient forwarding: We note that regardless of stock levels and the distance to the nearest private pharmacy, a large portion of patients (>60%) obtained private drugs. This could be because the drugs needed by patients are not stocked by the public system.^30^ Many drugs for non-communicable diseases, for example, are not on the essential drug list.^31 32^ Assessing whether these drugs are at the root of the bypassing behaviour could inform the inclusion of additional generic drugs to be routinely stocked by public facilities.

Another explanation is that dispensing behaviour causes patients to purchase private drugs. In primary facilities, providers may prescribe, but not dispense drugs, either to free up time to treat other patients or because they make money from private drug purchases.^26^ In hospitals, pharmacist shortages and absenteeism could mean pharmacies are not open to dispense drugs.

Third, drugs may be prescribed by the branded name only, despite national and state guidelines to prescribe generics.^21 33^ Since public facilities typically do not stock branded drugs, this would require patients to purchase private drugs. In clinical vignette data, most drugs prescribed in Odisha were identified by the brand name.^34^ The practice of prescribing branded drugs has been linked with a lack of provider knowledge of generic names, incentives from pharmaceutical companies and provider concerns that generic prescriptions put drug selection in the hands of unqualified pharmacists.^21 25 26^ A thorough investigation into exactly what is prescribed and dispensed is a key future direction for understanding public patient forwarding to private pharmacies.

### Policy implications

We focused on the state of Odisha, but, given that public patient forwarding is a phenomenon across India, we believe our analysis has implications for national and state policies. Our analysis suggests investing in the public supply of drugs, particularly in hospitals, could produce a minor reduction in financial hardship due to healthcare costs. Increasing average hospital drug stocks by 10 percentage points was associated with a reduction in CHE due to drugs of 3.7 percentage points (p=0.033), although this was not robust to our most extensive set of controls. Both evidence of ‘shopping’ among public hospital patients and the lower satisfaction ratings among primary facility patients obtaining private drugs suggest that the private drug preference is not absolutely dominant—Indian consumers are more open to drugs from public facilities than has been previously suggested.^17^

But there is the question of which policies could boost drug stocks. First, more public investment in the health sector could be considered. The Indian government invests less in health, at 0.9% of Gross Domestic Product (GDP), than other lower-middle-income countries (2.4% of GDP),^35^ and only a small share is allocated to drugs.^36^ Second, staff are not specifically incentivised to keep drugs in stock, despite the effort required to assess stocks and submit orders.^8^ Designing staff payment systems that incentivise drug stocks is thus another potential option, although this would also require investment in monitoring. Third, considering expanding the essential medicine list to drugs not currently available, including for non-communicable diseases (NCDs), is an option.^30^,^32^ Fourth, improving the management of drug supply could be considered.^8^ Rigid line-item budgets,^37^ and long administrative processes prohibit flexibility and responsiveness to demand.

An alternative set of policies concerns tapping into the vast network of private retail pharmacies—more than 850 000 private retail drug shops are in operation across India.^38^ Some of the issues that plague the public supply system are not in place in the private sector. For instance, since private pharmacies are paid per unit sold, they are incentivised to keep drug stocks high. Extending insurance programmes with appropriate provider payment methods to empanel private pharmacies could change such incentives as well as boost medicine access while minimising financial hardship for patients. If patients could freely use their insurance coverage to select medications from either public or private pharmacies, this could drive public facilities to compete against private pharmacies and maintain adequate drug stocks. However, such a programme would require a great extension of insurance scheme administration, including empanelling hundreds of thousands of private pharmacies. Furthermore, insuring drug costs risks exacerbating overuse of pharmaceuticals,^34 39^ which could fuel the ongoing crisis of irrational prescriptions, polypharmacy and antimicrobial resistance in India.^40^ Finally, the complex links between public and private sectors that lead to patient forwarding would need to be addressed through provider payment reforms to align incentives, address the quality of drugs being supplied and effectively enforcing regulations which are difficult tasks especially in the context of strong provider interest and low state capacity. Overall, policymakers should thus consider carefully how they should best engage the private sector, balancing the patients’ persistent desires for private drugs against the potential negative outcomes. More research targeted at how such policies would operate and their impact would be needed for state and national governments to consider such an approach.

Further expanding the provision of (cheaper) generics in standalone retail pharmacies is also an option.^9^ Retail shops that sell generics, Jan Aushadhi, have been established with the support of the government. Investigating their role in reducing CHE is another future research direction to consider.

Finally, depending on the results of future research, interventions that change provider prescription practices may be warranted. There are already guidelines in place that mandate providers to prescribe drugs by their active ingredients and not brand names. However, these are not strictly followed. Incentives for prescribing generics, for example, could be built into the extension of insurance coverage to drugs. Training programmes or guides regarding the names of generic drugs and their relationship with branded drugs could combat a lack of provider knowledge. Regulation or better enforcement of existing restrictions on pharmaceutical companies interacting with providers may also be an approach that reduces the prescription of branded drugs.

### Limitations

We note some limitations in our data and analysis. First, despite surveying a large number of households, we were limited by the small number of facilities and patients that could be linked to our sample. Because of the small sample sizes, some of our results were not robust to all controls. Second, none of our analyses are causal, so we must draw inferences from associations that could be affected by omitted variable bias, reverse causation or other issues. Third, we used data linked by distance and patient recall of the facility visited—patients may misremember or inaccurately identify the facility. We noted that the full sample and the linked samples are different across some dimensions. While we control for these factors in our analysis, we cannot rule out that they affect our findings. Some facilities were surveyed more than 30 days after the patient interview, and conditions at the facility could have changed since the visit, introducing random error which could have attenuated our estimates. Fourth, our outcomes are primarily drawn from patient self-report. Fifth, our patient satisfaction analysis does not directly test patient preferences. Eliciting patient preferences, expectations or beliefs about private drugs and directly testing the link between these patient characteristics and their choice of facility and drug type is an important future direction. Despite the limitations described, we believe our analysis is unique and rigorous in deploying a linked data set to assess the drivers of drug-driven financial hardship.

## Conclusions

Financial hardship due to drug costs is a nationwide problem in India that persists despite the large domestic manufacturing sector, price controls and the provision of free drugs in the public sector in most states. In this study, we showed there are multifaceted reasons for public patient forwarding to private pharmacies. A multipronged approach—one that addresses the role of the private retail market, public facility drug stocks, patient preferences and provider prescribing practices—will be required to reduce financial hardship and improve India’s health system performance in the area of financial protection.

## supplementary material

10.1136/bmjgh-2024-017788 online supplemental file 1

10.1136/bmjgh-2024-017788 online supplemental file 2

## Data Availability

Data are available upon reasonable request.
