# Supplementary material for: Public patient forwarding to private pharmacies: an analysis of data linking patients, facilities and pharmacies in the state of Odisha, India
Source: BMJ Glob Health. 2025 Feb 18;10(2):e017788. doi: 10.1136/bmjgh-2024-017788 (PMC11836861; doi:10.1136/bmjgh-2024-017788)
Supplement: online supplemental file 1 [file bmjgh-10-2-s001.docx]

# Reflexivity Statement

This study involved a partnership between researchers based in the US and India. The study addresses a key local research and policy priority – financial risk protection – as evidenced by India’s high rates of catastrophic health expenditure and recent efforts to reduce this outcome via the introduction of nationwide and state-based insurance programs. Local researchers are included as co-authors on the paper (AC and KG from Oxford Policy Management India; BB and AK are Indian nationals) that were involved in data collection, research design, and manuscript revision, including interpretation of the data. Project funds supported the work of these individuals to manage data collection and anlayze the data and these individuals have access to the data used in this study. This study will be disseminated through the broader project under which this work was conducted: the India Health Systems Reform Project, which is ongoing and engages policy-makers and researchers across India in trainings and study findings. Early career researchers, including currently enrolled students and recent graduates, are included as co-authors. All but one of the co-authors is female.
