# Supplementary material for: Public patient forwarding to private pharmacies: an analysis of data linking patients, facilities and pharmacies in the state of Odisha, India
Source: BMJ Glob Health. 2025 Feb 18;10(2):e017788. doi: 10.1136/bmjgh-2024-017788 (PMC11836861; doi:10.1136/bmjgh-2024-017788)
Supplement: online supplemental file 2 [file bmjgh-10-2-s002.pdf]

Appendix

Title: Public patient forwarding to private pharmacies: An analysis of data linking patients, facilities and pharmacies in the state of Odisha, India.

Table of Contents

Section	Page
Section 1: Additional Methodological Details	3
A: Household Sampling*	3
B: Weights Construction*	8
C: Facility Survey Sampling	10
D: Pharmacy Sampling	13
E: Mapping of facility types*	14
F: List of pharmaceuticals surveyed with direct observation by facility type/private retail pharmacy*	14
Section 2: Supplementary Results	18
G: Supplementary Tables and Figures	18
References	43

* Previously reported in the appendix for: Haakenstad A, Kalita A, Bose B, Cooper JE, Yip W. Catastrophic health expenditure on private sector pharmaceuticals: a cross-sectional analysis from the state of Odisha, India. Health Policy Plan. 2022 Aug 3;37(7):872-884.

List of supplementary tables and figures

	Page
Section 1: Additional Methodological Details	3
Table A.1: Tertile Positions of the 30 districts in Odisha based on Principal Component Analysis	4
Table A.2: Number of blocks required from the sampled districts (based on Proportionate Stratified Sampling), and the blocks sampled (based on Probability Proportional to Size)	5
Table A.3: Number of PSUs needed from the sampled districts (based on Proportionate Stratified Sampling), and the blocks sampled (based on Probability Proportional to Size)	6
Table A.4: Sample sizes for households based on screening criteria	7
Table C.1: Facilities covered for public hospitals providing secondary and tertiary level care	11
Table C.2: Facilities covered for public hospitals providing primary level care	12
Table D.1: Sample covered under chemists survey	13
Table E.1: Facility name and the level categorized to	14
Table F.1: Drugs surveyed for availability on the day of the survey	14
Section 2: Supplementary Results	18
Table G.1: All and linked outpatient visits	18
Table G.2: All and linked public facilities	20
Table G.3: All versus linked public hospital and primary facility outpatient users	22
Table G.4: All versus linked hospitals, primary facilities and private retail pharmacies	24
Figure G.1: Outpatient visits among primary facilities	25
Figure G.2: Out-of-pocket drug costs according to whether patients obtained some private drugs versus obtained only public drugs	25
Table G.5 Results from linear probability model of share of drugs in stock by hospitals and primary facilities (Table 3)	26
Table G.6: Results from linear probability model of private drugs and catastrophic health expenditure (CHE) in hospitals (Table 1)	27
Table G.7: Results from linear probability model of number of drugs and pharmacy referrals in hospitals (Table 1)	28
Table G.8: Results from linear probability model of private drugs and catastrophic health expenditure (CHE) in primary facilities (Table 1)	30
Table G.9: Results from linear probability model of number of drugs and pharmacy referrals in primary facilities (Table 1)	32
Table G.10: Sensitivity analysis of outcomes included in Table 1	34
Table G.11: Regression of patient rating of excellent or good needs met on private drugs obtained and any drugs obtained (Table 2)	36

Table G.12: Regression of patient rating of excellent or good quality on private drugs obtained and any drugs obtained (Table 2)	37
Table G.13: Sensitivity analysis of patient ratings among primary facilities, stratified by distance to the nearest pharmacy	39
Table G.14: Sensitivity analysis of outcomes included in Table 2	42

Section 1: Additional Methodological Details

A: Household Sampling

This section describes the complex multi-stage clustered sampling design adopted for selecting the households for our survey. The multiple hierarchical levels of clusters of the household sample were as follows:

1. District
2. Block
3. Villages in rural areas or enumeration blocks in urban centers—referred to as primary sampling units or PSUs
4. Household

Selection of Districts

The first level of the household sample was the district. Odisha has 30 districts that are classified under three administrative regions, Regional Development Clusters (RDC), each containing 10 districts. The three RDCs in the state are North, South and Central. Each RDC has differences in demographic and socio-economic characteristics such as poverty levels, proportion of tribal population, proportion of urbanization and industrialization. Therefore, districts were stratified by RDCs.

The district selection was done using a multi-step approach that aimed to represent all sections of the socio-economic, demographic, and geographical characteristics of the state. The following variables were considered for assignment of a “development” index to each district:

1. Population in the district
2. Percentage of urban population
3. Percentage of tribal population
4. District Gross Domestic Product (DGDP)
5. Level of poverty
6. Level of female literacy

Principal Component Analysis (PCA) was conducted for all 30 districts of the state with the above-mentioned six variables. The first Principal Component (PC1) was used to divide the 30 districts into tertiles as this component explained ~50 percent of the total variability in the data. The districts were then stratified by the three RDCs and tertiles within their respective stratum (RDC) (Table A.2).

Two districts from each RDC – one with a high tertile and the other with a low tertile – were selected through with-replacement random sampling. The randomization was through a computer-generated algorithm, and the process was repeated until we obtained a unique list of two districts with different tertiles under each RDC. With-replacement random sampling was chosen over without-replacement, as the former ensures that each district has an equal probability of being sampled.

From this process, we selected the following six districts:

- North RDC: Jharsuguda and Keonjhar
- South RDC: Kalahandi and Rayagada
- Central RDC: Balasore and Khorda

Table A.1: Tertile Positions of the 30 districts in Odisha based on Principal Component Analysis

	Northern Division		Central Division		Southern Division	
	Districts	Original tertile position	Districts	Original tertile position	Districts	Original tertile position
Division-wise Tertile 1						
	Angul	1	Cuttack	1	Boudh	2
	Dhenkanal	1	Jagatsingpur	1	Ganjam	2
	Jharsuguda	1	Khordha	1	Kalahandi	3
	Sambalpur	2	Puri	1	Kandhamal	3
Division-wise Tertile 2						
	Bargarh	2	Bhadrak	1	Koraput	3
	Sonepur	2	Jajpur	1	Nuapada	3
	Sundergarh	2	Kendrapara	1	Rayagada	3
Division-wise Tertile 3						
	Balangir	2	Balasore	2	Gajapati	3
	Deogarh	3	Mayurbhanj	3	Malkangiri	3
	Keonjhar	2	Nayagarh	2	Nabarangpur	3

Our sample yielded districts with a range of different characteristics. Among the sampled districts (highlighted in Table A.2), Jharsuguda, Kendujhar, Kalahandi, and Rayagada have a high concentration of Scheduled Tribe (ST) population; except Jharsuguda, all other districts are predominantly rural; Jharsuguda and Kendujhar have mining industries while the other districts are largely agricultural; Kendujhar, Balasore, Kalahandi, and Rayagada have high poverty levels. This distribution of characteristics reflect the diversities of the state to a large extent.

Selection of Blocks

The second level of clusters for the sample was the 'block,' which is the sub-district administrative unit in India.[1] A total of 30 blocks were selected from the six sampled districts. To select blocks, the following methods were used:

1. First, to determine the number of blocks to be chosen from each district, the method of Proportionate Stratified Sampling (PSS) was used —the district being the stratum and the proportion determined by the population of the district. Based on this, districts with bigger population sizes contributed a greater number of blocks to the sample than districts with smaller populations.
2. From each district, the blocks were sampled using Probability Proportional to Size (PPS).35 This ensured that the larger the population size of the block, the greater the chances of its inclusion in the sample.

First, the cumulative population of all blocks in each district was calculated. Then the total population of the district was divided by the sample size of blocks required from that district (Table A.1). This number

[1] On an average, a district comprises of 10 blocks. The average population of a district is 1,000,000, and of a block is 100,000. There is wide variation among districts and blocks across the country as well as within Odisha.

generated the Sampling Interval (SI). A computer-generated Random Start (RS) number between zero and the Sampling Interval was used. The population boundaries of the block that contained the RS number was selected as the first block (Populationblock>RS). For selecting the subsequent blocks, the RS number was added to multiples of the Sampling Interval, the second block was selected by Populationblock>(RS+1SI), the third block by Populationblock>(RS+2SI) and so on, till the required number of blocks from each district were sampled.

Table A.2: Number of blocks required from the sampled districts (based on Proportionate Stratified Sampling), and the blocks sampled (based on Probability Proportional to Size)

S.No.	Sampled Districts (as per PCA)	Total blocks	Number of blocks to be sampled (as per PSS)	Blocks sampled
1.	Kendujhar	13	6	1. Anandapur<br>2. Ghasipura<br>3. Harichandanpur<br>4. Jhumpura<br>5. Joda<br>6. Saharapada
2.	Balasore	12	8	1. Balesore<br>2. Baliapal<br>3. Basta<br>4. Bhogarai<br>5. Jaleswar<br>6. Nilagiri<br>7. Remuna<br>8. Soro
3.	Khorda	10	5	1. Balipatna<br>2. Begunia<br>3. Bolagad<br>4. Jatni<br>5. Tangii
4.	Rayagada	11	3	1. Bisam Cuttack<br>2. Kaashipur<br>3. Padmapur
5.	Kalahandi	13	5	1. Bhawanipatna<br>2. Golamunda<br>3. Kalampur<br>4. Koksara<br>5. Narla
6.	Jharsuguda	5	2	1. Jharsuguda<br>2. Lakhanpur

Selection of Primary Sampling Units

Primary Sampling Units (PSUs) were the third level of clusters to be sampled. PSUs of each block were stratified into urban and rural (based on census data). 'Rural' is defined as census-villages, and 'Urban' is defined as census enumeration blocks.

Based on the proportion of rural and urban population in the state, the rural-urban ratio for the sample was determined as 85:15. The sample contained approximately 85 percent rural PSUs and 15 percent urban PSUs. A total of 375 PSUs were sampled from the 30 blocks – 300 villages (rural) and 75 enumeration blocks (urban).

Sampling of PSUs was done using the same methods that we used for sampling blocks. First, Proportionate Stratified Sampling (PSS) was used to determine the number of PSUs that each block would contribute to the sample, calculated based on their population size. 85 percent of this number

was the required number of rural PSUs and 15 percent was for urban PSUs. Compared to less populous blocks, more populous blocks contributed a greater number of PSUs to the sample.

Next, once the number of PSUs to be sampled from each block was determined, Probability Proportional to Size (PPS) was used to sample the PSUs in each block. The larger the size of the PSU, the greater the chance of its inclusion in the sample.

The cumulative population of all PSUs in each block was calculated. Then the total population of the block (ΣpopulationPSUs) was divided by the sample size of PSUs required from that block (Table A2.5). This number generated the Sampling Interval (SI). A computer-generated Random Start (RS) number between zero and the SI was used. The population boundaries of the PSU that contained the RS number was selected as the first PSU (PopulationPSU>RS). For selecting the subsequent PSUs, the RS number was added to consecutive multiples of the SI, as in the second PSU was selected by PopulationPSU>(RS+2SI), the third PSU by PopulationPSU>(RS+2SI) and so on, until the required number of PSUs from each block were sampled. The process was repeated till the sample included the required numbers of rural and urban PSUs.

Table A.3: Number of PSUs needed from the sampled districts (based on Proportionate Stratified Sampling), and the blocks sampled (based on Probability Proportional to Size)

District	Block	PSU rural	PSU urban	PSU total
Baleswar	Balesore	13	11	24
	Baliapal	10	0	10
	Remuna	9	3	12
	Bhograi	15	0	15
	Jaleswar	10	2	12
	Basta	10	0	10
	Nilagiri	7	1	8
	Soro	7	3	10
Total		81	20	101
Jharsuguda	Lakhanpur	26	6	32
	Jharsuguda	15	4	19
Total		41	10	51
Khorda	Balipatna	6	2	8
	Begunia	7	0	7
	Bolagad	7	0	7
	Jatni	5	6	11
	Tangii	8	1	9
Total		33	9	42
Kalahandi	Bhawanipatna	19	19	38
	Golamunda	15	0	15
	Jaipatna	15	2	17
	Kalampur	7	0	7
	Koksara	13	0	13
	Narla	14	0	14
Total		83	21	104
Rayagada	Bisam Cuttack	3	2	5
	Kaashipur	5	0	5
	Padmapur	2	0	2
Total		10	2	12
Kendujhar	Anandapur	7	3	10
	Ghasipura	10	0	10
	Harichandanpur	10	3	13
	Jhumpura	8	3	11
	Joda	11	4	15
	Saharapada	6	0	6
Total		52	13	65

Selection of Households

To sample households, first, all households in each sampled PSU were listed. From the listing data, the following information about each household in a PSU was collected: (i) Number of households with no event (NE); (ii) Number of households with an outpatient visit in the last 15 days (O); (iii) Number of households with a chronic illness diagnosed by a health provider (C); and (iv) Number of households with hospitalization in the last one year (H).

In addition to the four points of information above, the listing tool also collected information on the 'preferred provider of the household for outpatient care' and 'preferred provider of the household for inpatient care'. While this information was not used for household sampling, this was used for provider sampling (described later in this report).

An absolute precision of 0.007 and a design effect of 2.5 were assigned to arrive at a household sample size of 7500. Considering the rarest event, hospitalization (0.04 or 4.4 percent based on NSSO data), for a sample of 7500 households, the confidence interval (CI) is 95 percent and design effect is 2.5. The number of households sampled from each category were 3000 for no event (NE), and 1500 households each from the outpatient visit (O), chronic illness (C) and hospitalization (H) categories. The oversampling of these latter categories (O, C and H) in comparison to the NE group aims to provide enough precision to stratified estimates of these sub-samples, such as disease-specific expenditures or gender-specific care-seeking frequency. These sample sizes were based on the highest possible number of households that could have these events, as well as the margin of error estimates for variables of interest, for example, expenditure per hospitalization, expenditure per outpatient visit, or percentage referred to chemists. Therefore, the overall margin of error for the estimates of these groups grew even smaller, ranging from 0.000 to 0.001 percent.

The total number of households sampled was 7567. Data was collected about each member of each of these households, so the total number of individuals in the sample was approximately 30,645.

Table A.4: Sample sizes for households based on screening criteria

Category	Maximum possible in the universe (based on NSSO & NFHS estimates)	Sample size (based on margin of error estimates for variables of interest)
Hospitalization - H (last one year)	3,655	1500
Outpatient visit – O (last two weeks)	2,442	1500
Chronic illness (C)	6,105	1500
No event (includes illness but none of the above three categories, and no illness) (NE)	70,298	3000
**Total**	82,500	7500

The number of households to be selected from each PSU was fixed at 20. PPS gives us an unequal probability for selection of districts, blocks, and PSUs that results in higher clusters based on their population sizes. However, selection of the same number of elements from unequally sized clusters neutralizes that unequal probability of selection, leading to self-weighting of the sample and equal probability for selection of households. Therefore, 20 HH from each PSU was fixed in order to maintain the integrity of the sample.

To select the 20 households from each PSU, all the households in the PSU were first categorized into the four categories shown above – NE, O, C, H based on the household listing data. These were randomly ordered into MS Excel. The ratio of 2:1:1:1 was followed (based on the sample sizes of 3000 for NE and 1500 each for O, H and C). So, out of the 20 households in each PSU, eight were in NE, and four were

from each of the other three categories – O, C, H (not exclusive, some of the households had a combination of these three events).

- Out of the randomly ordered households, the first eight households falling under NE were selected (this included households with no illness in the last 15 days, illness in the last 15 days but none of the other three events, i.e., no chronic illness, no hospitalization, no outpatient visit). This ensured our sample included households that did not access care (foregone care or self-treatment), as well as households that did not have any illness in the last 15 days.
- Out of the randomly ordered households, the first four households that had said "yes" to an outpatient visit in the last 15 days were selected (irrespective of whether they had said "yes" to chronic illness and hospitalization).
- Out of the randomly ordered households, the first four households that had said "yes" to hospitalization in the past one year were selected (again, irrespective of whether they had said "yes" to chronic illness and outpatient visit).
- Out of the randomly ordered households, the first four households that had said "yes" to having been diagnosed with a chronic illness were selected (again, irrespective of whether they had said "yes" to chronic illness and outpatient visit).

There were eight possible categories that came up for households:

1. Households with no event (NE)
2. Households with a diagnosed chronic illness (C)
3. Households with an outpatient visit in the last 15 days (O)
4. Households with an inpatient visit/hospitalization in the past one year (H)
5. Households with an outpatient visit in the last 15 days and a diagnosed chronic illness (C+O)
6. Households with a hospitalization in the past year and a diagnosed chronic illness (C+H)
7. Households with a hospitalization in the past year and an outpatient visit in the last 15 days (O+H)
8. Households with a hospitalization in the past year and an outpatient visit in the last 15 days and a diagnosed chronic illness (C+O+H)

B: Weights construction

Sampling weights were computed for the household survey to take into account the multistage sampling strategy and the oversampling of certain types of households. Weights account for three dimensions:

(1) Selection of households in each village (PSU/cluster) based on four household characteristics;
(2) Selection of each village (PSU/cluster) and block (strata) based on sampling proportionate to the size of the population; and
(3) Selection of districts based on a development index.

(1) Selection of households in each village (PSU/cluster) based on four household characteristics

A listing of each selected village was conducted in which each household was asked whether household members had any of the following:

1. Case of hospitalization in the past year

2. Case of chronic illness
3. Case of outpatient care in the past two weeks
4. None of the above – categorized as a no illness household

In each village, 20 households were selected. For conditions a)-c), 4 households were selected at random to participate in the survey. Among households with no illness, d), 8 households were selected. However, many households had more than one of these conditions, making them eligible to be selected for more than one group. Therefore, we extended the probability of selection to take into account intersections represented by four additional categories:

5. Case of hospitalization and chronic illness
6. Case of outpatient care and chronic illness
7. Case of outpatient care and hospitalization
8. Case of outpatient care, hospitalization and chronic illness

Let each of these conditions be denoted by j. Probability of j or P(j) then is given by:

$$P(j) = \frac{k_{ji}}{K_i} \ \forall\, j \ \in \{1,2,3,4,5,6,7,8\}$$

where {1,2,3,4, 5,6,7,8} are one of the eight conditions, k_{ji} is the number of households with these conditions that participated in the survey and K_{ji} is the number of HH with condition 'j' in cluster 'i'

(2) Selection of each village (PSU) and block based on sampling proportionate to the size of the population

Blocks and PSUs were selected based on sampling proportionate to the size of the population. The probability proportionate to size (PPS) based sampling ensures that more populous clusters have a high probability of selection. However, as the same number of individuals are sampled from each cluster, individuals in a larger cluster have a lower probability of selection, which is taken account in step (1). Therefore, in step (2), we calculate just the probability of selection for blocks and PSUs.

Let P_P be the weighted probability of each cluster being selected – this is calculated based on the number of households in the PSU as a share of all households in the block. Finally, each block in a district has a probability P_B of being selected based on the size of the population in the block divided by the population of all blocks in the district. The PPS weights are thus given by:

$$PPS\ weight = \frac{1}{(P_B * P_P\ )}$$

Combining these probabilities with the probability from the household conditions, the base weight (BW) for the household survey is given by:

$$BW = \frac{1}{(P_B * P_P * \ P(j))}$$

(3) Selection of districts based on the development index of population, female literacy, poverty and gross domestic product, and social groups.

A development index of population, female literacy, poverty and gross domestic product, and social groups was calculated to categorize districts by development status and select districts based on these strata in addition to geography. We address this selection approach by calibrating our Base Weights to the known population totals of the factors in the development index for the state of Odisha. We use the method of iterative proportional raking to construct these post-stratification weights.[1] This method minimizes the differences between known population totals and the survey in an iterative manner, raking the distribution in the survey to the known population totals one margin at a time until the differences are minimized across all margins. An adjustment factor (π) is thus applied to the Base Weights (BW) to produce Raked Weights that represent these population totals.

The final Raked Weight (RW) is thus:

$$RW = BW * \pi$$

We relied predominately on the 2011 Indian census of Odisha to calculate the known population totals.[2] We raked over three margins: 1) the distribution of households by social group (Scheduled Tribe, Scheduled Caste and Other) and rural versus urban residence; 2) the distribution of households with at least one female matriculate (10th grade or higher) by rural and urban residence; 3) the distribution of households with one member above the age of 60; and 4) the share of the population living below the poverty line, based on the population totals from the Reserve Bank of India and the reported possession of a Below Poverty Line card by households in the survey.[3]

C: Sampling for Facility Surveys

Our sample size for facility surveys is 554 healthcare facilities in the six sampled districts in Odisha. These included healthcare facilities across all levels of care – primary, secondary and tertiary, in both the public and private sectors. The following sections describe the sampling methodologies that we used for the different categories of facilities.

Sampling for facilities providing secondary and tertiary care

The survey of facilities providing secondary and tertiary level care (hospital survey) aimed to cover both the public and private facilities. Our target was a census of all four Medical College Hospitals in the sampled districts. In addition, the All India Institute of Medical College (AIIMS) and Capital Hospital in Bhubaneshwar, two large tertiary care hospitals were included, resulting in a targeted sample size of six Medical College and Tertiary Hospitals. For District Hospital (DH), Sub-Divisional (SDH) Hospital, Community Health Centers (CHC), our target was a census at the district level across all the six sampled districts. Public hospitals include Medical College Hospitals, District Hospitals, and Sub-Divisional Hospitals. “Other” hospitals consist of first referral units, municipal hospitals, and other hospitals such as those run by the Ministry of Railways, the Employees State Insurance Corporation (ESIC), and other government departments or programs. Our targeted samples sizes for each of these categories of facilities were six DHHs, five SDHs, and 84 CHCs. In addition, 31 “Other” public hospitals listed on the state government website was included. Around 63 private hospitals, nursing homes and clinics that are in the state were also included based on the inclusion criteria. Single specialty facilities were excluded.

Public sector facilities for secondary and tertiary care
Public sector health facilities for secondary and tertiary care included Medical College Hospitals, District Hospitals (DH), Sub-Divisional Hospitals (SDH) and Community Health Centers (CHC) under the government's health delivery system in Odisha. All the MCHs in the state, along with AIIMS-Bhubaneswar and Capital Hospital (Bhubaneswar) were included in the sample. All the DHs, SDHs, CHCs and "other" hospitals in the six sample districts were part of the sample.

The total sample covered under the hospital and CHC survey (Survey 2 in Table A2.1) is given below in Table A2.7. In total, the survey collected data from 122 hospitals – six Medical College Hospitals, four DHH, five SDH, 83 CHC and 29 "other" hospitals. These sample sizes fell short from the targets (shown in Table A2.7). Two MCHs and two DHs did not provide consent to participate in the study. For AIIMS-Bhubaneswar, the survey could not be completed due to COVID-19. Among the facilities in the "others" category, consent was provided by only 29 facilities.

Table C.1: Facilities covered for public hospitals providing secondary and tertiary level care

S. No	District	MCH	DHH	SDH	Others	CHC
1	Jharsuguda		1 (1)		1 (1)	6 (6)
2	Kendujhar		0 (1)	2 (2)	6 (6)	16 (17)
3	Balasore	0 (1)	0 (1)	1 (1)	1 (1)	17 (17)
4	Khorda	1 (1)	1 (1)		17 (20)	16 (16)
5	Kalahandi		1 (1)	1 (1)	2 (2)	17 (17)
6	Rayagada		1 (1)	1 (1)	2 (2)	11 (11)
	Other districts	5 (7)				
	**Total**	**6 (9)**	**4 (6)**	**5 (5)**	**29 (31)**	**83 (84)**
Note: Target number of facilities are provided in parentheses						

The primary respondent for these health facilities was the Officer-in-Charge, (permanent or temporary/acting), such as Director or Chief Executive Officer, Medical Superintendent, Hospital Manager, Administrative Officer, Chief Medical Officer. In some cases, the interviewer needed to interview more than one respondent for each facility, since one single respondent did not have all the information.

Private sector facilities for secondary and tertiary care
The sampling frame for the private sector facilities providing secondary and tertiary level care included private hospitals and nursing homes, excluding single-specialty facilities. In the absence of a comprehensive central database providing the details of all private facilities, the database was compiled by collecting information from various secondary sources: (i) Lists of empaneled hospitals with the

government sponsored health insurance schemes (RSBY, BSKY, BKKY); (ii) Information from Chief Medical Officers/Chief Health Officers in districts and Block Medical Officers in blocks; (iii) Lists of providers maintained by the Indian Medical Association/Medical Council of India and Hospital Administrators Associations; and (iv) Information from the household listing exercise that stated preferred providers for inpatient care. Based on a combination of these information, a list of 63 private hospitals located in the six sample districts was compiled and approached for data collection. Out of these, 36 hospitals gave consent to participate in the survey.
The primary respondents for these health facilities were the Officers-in-Charge, such as Director or Chief Executive Officer, Medical Superintendent, Hospital Manager, Administrative Officer and Chief Medical Officer.

Sampling for facilities providing primary care
The sample for our survey on primary care facilities included Primary Health Centers (PHC), Sub-centers (SCs) and Health Wellness Centers (HWCs) under the public health system in the state (Survey 3 in Table A2.1)

Table C.2: Facilities covered for public hospitals providing primary level care

S. No	District	PHC	SC	HWC
1	Jharsuguda	5	34	2
2	Kendujhar	24	52	3
3	Balasore	45	75	0
4	Khorda	26	33	1
5	Kalahandi	16	63	1
6	Rayagada	13	10	0
	**Total**	**129**	**260**	7

For primary care facilities, our target was to undertake a census of all PHCs, SCs and HWCs in the sampled blocks. In the 30 sampled blocks, there were a total of 158 PHCs and 375 SCs. The data from the Department of Health and Family Welfare about HWCs in Odisha was not found to be up to date. On paper, the number of HWCs listed for the blocks in our study sample was 70 (at the time of sampling in June-August 2019), but only seven HWCs were found to exist.

There is a shortfall in the number of facilities covered in the survey compared to the target sample sizes. For PHCs there was a shortfall of 28 facilities, out of which two were converted into HWCs, 22 were converted into CHCs but had not been updated on the official database, and four were not functional at the time of our data collection due to the effects of the cyclone in Odisha during May 2019. Of the 39 Sub-centers, three SCs did not have any staff available and one SC served two PSUs under our study.

D: Sampling for solo providers and chemists

The respondents for the survey on chemists included shopkeepers and shop-owners of private pharmacies or drug stores. It should be noted here that we did not use any criteria of medical qualifications to identify chemists. Our sample could, therefore, include both medically qualified pharmacists, as well as unqualified pharmacists.

Since the universe for chemists is not known, the following strategies were used to arrive at the sampling frame in the six sampled districts:

1. Mapping of chemist shops within a three kilometer radius of each surveyed facility
2. Information gathered from respondents of facility surveys and chemist surveys through snowball sampling
3. Using the household listing data, where the households were asked about the service provider they normally visit for outpatient care or pharmacies

Table D.1: Sample covered under chemists survey

S. No	District	Pharmacists
1	Jharsuguda	67
2	Kendujhar	200
3	Balasore	272
4	Khorda	197
5	Kalahandi	200
6	Rayagada	100
	**Total**	**1036**

The targeted samples sizes were 1000 for chemists across the six districts. Against these targets, the study achieved a sample of 1036 chemists.

E: Mapping of facility types

Table E.1: Facility name and the level categorized to

#	Facility name	Level
1.	Medical College and tertiary hospital	Public Hospital

2.	District Hospital/Municipal Hospitals (urban)	Public Hospital
3.	Sub-District Hospital	Public Hospital
4.	Community Health Center/First Referral Unit/Rural Hospital (CHC/FRU)	Public Hospital
5.	Urban Health Center/Urban PHC/Urban Health Post	Public Primary
6.	Primary Health Center (PHC)	Public Primary
7.	Sub-Center/Health and Wellness Center (SC/HWC)	Public Primary
8.	ASHA (Community Health Worker)	Public Primary
9.	Anganwadi Center	Public Primary
10.	Mobile Medical Unit	Public Primary
11.	Health Camp	Private Primary
12.	All other facility types	N/A

F: List of pharmaceuticals surveyed with direct observation by facility type/private retail pharmacy

Table F.1: Drugs surveyed for availability on the day of the survey

**Product**
AMITRIPTYLINE CHLORDIAZEPOXIDE*
AMOXYCILLIN
AMPICILLIN*
AMLODIPINE
VITAMIN C
ASPIRIN*

ATENOLOL
ATORVASTATIN*
CALCIUM GLUCONATE*
CARBAMAZEPINE
CETRIZINE
CHLOROQUIN
CHLORPHENIRAMINE MALEATE
CIPROFLOXACIN
COTRIMOXAZOLE
DEXAMETHASONE
DICLOFENAC
DICYCLOMINE
DOMPERIDONE
DOXYCYCLINE
ENALAPRIL
ERYTHROMYCIN
FERROUS SULPHATE AND FOLIC ACID
FOLIC ACID
FRUSEMIDE
GENTAMICIN
GLIMEPIRIDE*
GLIPIZIDE*
IBUPROFEN

ISOSORBIDE DINITRATE*
METFORMIN*
METRONIDAZOLE
NIFEDIPINE
ORS PACKET
PARACETAMOL
PENICILLIN G POTASSIUM
PHENOBARBITONE*
PHENYTOIN SODIUM*
PREDNISOLONE
RANITIDINE
RABIES VACCINE
SODIUM BI-CARBONATE
SALBUTAMOL*
SODIUM VALPROATE*
THEOPHYLLINE AND ETOFYLLINE
TRIHEXYPHENIDYL*
LEVO THYROXIN*
VITAMIN B COMPLEX
ZINC
NORADRENALINE*
ARTESUNATE
LIGNOCAINE*

MAGNESIUM SULPHATE*
OXYTOCIN
SURGICAL SPIRIT
TETANUS TOXOID
TINIDAZOLE
VITAMIN A
METHYLCOBALAMINE
NORFLOXACIN
OMEPRAZOLE

* Surveyed in hospitals and community health centres (CHCs) only.

Section 2: Supplementary Results

Table G.1: All and linked outpatient visits

	All Outpatients of public facilities	SE	Outpatients linked to public facilities	SE	P-value	
Linked with a facility	60%	-	100%			
Average age	33.3	0.9	32.5	1.2	0.214	
Share female	48%	2%	46%	2%	0.151	
Share living in a rural area	84%	3%	85%	3%	0.261	
Share belonging to a scheduled tribe	15%	2%	16%	3%	0.228	
Share belonging to a scheduled caste	18%	2%	18%	2%	0.863	
Share illiterate	25%	2%	25%	2%	0.459	
Share reporting insurance coverage	17%	2%	15%	2%	0.342	
Share reporting poor or fair health	22%	2%	20%	2%	0.045	*
Share reporting diagnosis of a chronic condition	24%	2%	20%	2%	0.004	**
Average household consumption expenditure (USD)	$1,928.1	73.8	$1,911.5	104.1	0.755	
Average cost per outpatient visit (USD)	$11.2	0.7	$9.0	0.7	<.001	***
Average drug cost per outpatient visit (USD)	$6.1	0.4	$5.2	0.3	0.004	**
Share of outpatient visits with any drug cost	77%	2%	77%	2%	0.965	
Share attending a CHC	43%	3%	56%	4%	<.001	***
Share attending a hospital	27%	2%	16%	3%	<.001	***
Share attending a PHC	29%	3%	27%	4%	0.311	
Share attending a HWC or SC	2%	1%	1%	<1%	0.201	

Share attending the closest government facility	78%	2%	84%	2%	<.001	***
CHE (10%) due to total outpatient care cost	25%	2%	22%	2%	0.005	**
CHE (10%) due to drug outpatient care cost	13%	1%	12%	2%	0.213	
Share using the private sector for drugs among those obtaining drugs	72%	2%	73%	3%	0.573	
Time (minutes) waited at facility	29.7	1.7	28.3	1.9	0.27	
Share using care for fever	58%	2%	64%	3%	<.001	***
Share using care for childbirth services	3%	1%	2%	1%	0.020	**
Share using care for diarrhea	3%	1%	3%	1%	0.416	
Share using care for injury	3%	1%	3%	1%	0.906	
Share using care for acute respiratory condition	8%	1%	9%	2%	0.194	
Share prescribed medicine	89%	1%	90%	2%	0.873	
Share referred to a particular pharmacy	14%	2%	14%	2%	0.654	
Share recommended an alternative drug at pharmacy	1%	<1%	<1%	<1%	0.383	
Share requesting an alternative drug at pharmacy	1%	<1%	0%	0%	0.082	
Share obtaining any drugs	89%	1%	90%	2%	0.873	
Average number of drugs obtained	2.9	0.1	2.9	0.1	0.383	
Share reporting good or excellent in terms of needs met at visit	81%	2%	79%	2%	<.001	***
Share reporting excellent or good quality of care at visit	74%	2%	74%	2%	0.807	
N	1530		917			

Table G.2: All and linked public facilities

	All Public Facilities	SE	Linked Public Facilities	SE	P-value	
Share Hospital	8%	1%	9%	2%	0.438	
Share CHC	16%	2%	29%	4%	0.000	***
Share PHC	25%	2%	34%	4%	0.004	**
Share HWC & SC	51%	2%	27%	4%	0.000	***
Total hours open per week	41.2	1.4	44.4	1.9	0.152	
Share providing inpatient care	23%	2%	43%	4%	<.001	***
Share reporting an electronic record-keeping of services	47%	2%	59%	4%	0.001	***
Average number of beds	14.0	4.9	32.5	16.1	0.019	*
Share in a rural area	91%	1%	87%	3%	0.098	
Share reporting having a flush toilet	55%	2%	63%	4%	0.018	*
Share reporting having a severe water shortage	30%	2%	23%	4%	0.021	*
Share reporting being always accessible	97%	1%	97%	1%	0.831	
Average distance to closest pharmacy (kilometers)	9.2	6.4	25.0	23.5	0.130	
Median distance to closest pharmacy (kilometers)	0.3		0.1			
Share with a pharmacy 500+ meters away	48%	2%	29%	4%	<.001	***
Average daily: outpatient visits among facilities / clients among pharmacies	70.2	4.8	105.4	10.1	<.001	***
Number of staff (present) / working at pharmacy	8.9	2.2	16.6	7.1	0.032	**
Share reporting computerized tracking of pharmacy stocks	22%	2%	36%	4%	<.001	***
Share using electronic ordering for drugs	15%	2%	20%	4%	0.024	**
Share reporting 2 or more weeks for a delivery to be received	23%	2%	17%	3%	0.056	
Share reporting mostly or always received fewer drugs than ordered	24%	2%	29%	4%	0.131	
Share reporting they never receive expired drugs	28%	2%	30%	4%	0.570	
Share of essential medicine list drugs in stock	39%	1%	50%	2%	<.001	***
Share reporting branded medicine among the top 5 drugs dispensed	10%	1%	8%	2%	0.419	

Share reporting generic medicine among the top 5 drugs dispensed	88%	1%	93%	2%	0.035	**
Share reporting fixed dose combination medicine among the top 5 drugs dispensed	4%	1%	3%	2%	0.811	
Share reporting antibiotics among the top 5 drugs dispensed	72%	2%	76%	4%	0.143	
Share of patients given advice to						
Share reporting ever suggesting alternatives						
Average share of patients to which alternatives are suggested						
Average share of patients obtaining branded drugs, as reported by pharmacists						
Average share of patients obtaining branded generic drugs, as reported by pharmacists						
Whether pharmacy a Jan Aushadhi Pharmacy						
N	523		143			

Linked versus overall samples

Over half of outpatient visits could be linked (Table G3). The full and linked patient samples were similar across most dimensions. However, the hospital outpatient sample differed in ways that indicate there was better linkages with CHCs, which could be due to patients using non-CHC hospitals outside of the sampled areas. The linked versus full hospital sample included fewer female patients (45% vs. 49%), fewer patients with poor or fair health (19% vs. 23%) and reporting a chronic condition (22% vs. 25%). They also spent less OOP ($9.8 vs. $13.0), were more likely to visit the closest government facility (82% vs. 75%), and use care for fever (66% vs. 58%) and childbirth (2% vs. 3%). In the primary facility outpatient sample, more rural patients were linked as compared to the full sample (97% vs. 93%).

As shown in Table G4, the linked and unlinked samples also differed in terms of the provision of inpatient care and whether the closest pharmacy was more than 500 meters away. Among primary facilities, the linked sample had a higher concentration of PHCs, which tend to be more advanced and better equipped facilities, explaining differences in terms of the share of facilities with water shortages, outpatients per day, staff and the share of essential medicines in stock between the linked and overall samples.

Table G.3: All versus linked public hospital and primary facility outpatient users

	All outpatients of public hospitals	SE	Outpatients linked to public hospitals	SE	P-value		All outpatients of public primary facilities	SE	Outpatients linked to public primary facilities	SE	P-value	
Linked with a facility	63%		100%				53%		100%			
Average age	33.7	1.1	32.8	1.4	0.254		32.5	1.4	31.8	1.7	0.587	
Share female	49%	2%	45%	3%	0.033	*	47%	3%	48%	4%	0.598	
Share living in a rural area	80%	4%	81%	4%	0.426		93%	2%	97%	1%	0.050	*
Share belonging to a scheduled tribe	13%	3%	15%	4%	0.191		19%	3%	20%	3%	0.637	
Share belonging to a scheduled caste	19%	2%	18%	3%	0.72		16%	2%	18%	4%	0.403	
Share illiterate	25%	2%	27%	3%	0.283		23%	2%	22%	3%	0.658	
Share reporting insurance coverage	18%	2%	17%	2%	0.493		15%	3%	13%	4%	0.438	
Share reporting poor or fair health	23%	2%	19%	2%	0.02	*	21%	3%	22%	4%	0.713	
Share reporting diagnosis of a chronic condition	25%	2%	22%	3%	0.025	*	21%	3%	16%	3%	0.083	
Average household consumption expenditure (USD)	$1981.5	94.4	$2011.2	137.8	0.627		$1808.8	117.0	$1656.3	129.8	0.116	
Average cost per outpatient visit (USD)	$13.0	1.0	$9.8	0.8	<.001	***	$7.4	0.7	$7.1	1.0	0.739	
Average drug cost per outpatient visit (USD)	$6.7 (6.3 in my paper)	0.5	$5.4	0.4	0.002	***	$4.9	0.4	$4.9	0.6	0.933	
Share attending a CHC	62%	3%	78%	4%	<.001	***						
Share attending a HWC or SC					.		6%	2%	3%	2%	0.261	
Share attending the closest government facility	75%	2%	82%	2%	<.001	***	86%	3%	90%	4%	0.154	
CHE (10%) due to total outpatient care cost	30%	2%	25%	2%	0.001	**	15%	2%	15%	3%	0.889	
CHE (10%) due to drug outpatient care cost	14%	2%	12%	2%	0.014	**	10%	2%	12%	3%	0.114	
Share using the private sector for drugs among those obtaining drugs	71%	3%	72%	4%	0.637		74%	3%	75%	4%	0.726	
Time (minutes) waited at facility	32.3	2.2	30.5	2.4	0.23		23.9	2.3	22.6	1.9	0.575	
Share using care for fever	58%	3%	66%	3%	<.001	***	58%	4%	61%	5%	0.433	
Share using care for childbirth services	3%	1%	2%	1%	0.009	**	2%	1%	2%	1%	0.865	
Share prescribed medicine	89%	2%	88%	2%	0.231		89%	3%	93%	2%	0.102	
Share referred to a particular pharmacy	14%	2%	14%	2%	0.757		13%	2%	16%	4%	0.225	
Share obtaining any drugs	89%	2%	88%	2%	0.231		89%	3%	93%	2%	0.102	
Average number of drugs obtained	3.0	0.1	2.9	0.1	0.145		2.8	0.1	2.9	0.1	0.633	
Share reporting good or excellent in terms of needs met at visit	80%	2%	78%	2%	0.141		83%	3%	81%	4%	0.587	
Share reporting excellent or good quality of care at visit	73%	2%	74%	3%	0.592		75%	3%	75%	4%	0.832	
N	1032		654				498		263			

Notes: Linked public facilities include all facilities to which a public outpatient visit was linked via the report of the facility by the patient in the household survey. All means and linearized standard errors calculated with the survey generated weights. Hospital users include outpatient visits at public hospitals and community health centres (CHCs). Primary facility users include outpatient visits at primary health centres (PHCs), health and wellness centres (PHCs) and sub-centres (SCs). Public Ayush were excluded because these providers were not surveyed at the facility level. SE: Standard Error. CHE: Catastrophic health expenditure calculated as 10% or more spent on health as a share of monthly household consumption expenditure. Statistical significance of difference in means represented by p-values: * <.05, ** <.01, ***<.001.

Electronic ordering of drugs occurred somewhat frequently in hospitals (37%) but infrequently among private retail pharmacies (3%) and primary facilities (6%). Even though private retail pharmacies used computerized methods of stock management and electronic ordering less frequently, they reported more timely delivery of stocks (0% reporting two weeks or more to receive orders versus >15% in public facilities); full realization of orders (2% mostly or always receiving fewer drugs than ordered vs. >20% in public facilities); and never receiving expired drugs (86% vs. >25% in public facilities).

Table G.4: All versus linked hospitals, primary facilities and private retail pharmacies

	All Hospitals	SE	Linked Hospitals	SE			All Primary Facilities	SE	Linked Primary Facilities	SE			All Private Retail Pharmacies	SE	Private Retail Pharmacies <500m	SE		
Share CHCs	67%	4%	76%	6%	0.059													
Share HWCs & SCs							67%	2%	44%	5%	0.000	***						
Share providing inpatient care	86%	3%	98%	2%	0.000	***	4%	1%	8%	3%	0.019	*						
Share reporting having a severe water shortage	18%	3%	20%	5%	0.586		34%	2%	24%	5%	0.029	*						
Average distance to closest pharmacy (kms)	0.3	0.1	0.1	0.1	0.035	*	2.9	0.2	1.9	0.3	0.002	**						
Share with pharmacy within 500 meters	84%	3%	95%	3%	0.003	**	42%	2%	56%	5%	0.003	**						
Average outpatient visits per day	201.8	14.3	211.5	17.8	0.549		31.3	2.2	41.3	4.3	0.016	*	44.8	1.5	47.7	2.2	0.020	*
Total number of staff (present)	30.0	9.1	38.3	18.0	0.410		2.5	0.1	3.0	0.3	0.004	**	2.1	0.0	2.2	0.1	0.038	*
Total number of pharmacists (present)	1.6	.17	1.8	.252	.163		0.3	<.1	0.5	<.1	.989							
Share reporting computerized tracking of pharmacy stocks	72%	4%	78%	6%	0.197		4%	1%	6%	3%	0.247		18%	1%	18%	2%	0.791	
Share using electronic ordering	37%	4%	35%	6%	0.560		6%	1%	10%	3%	0.090		3%	1%	2%	1%	0.344	
Share reporting 2 or more weeks for a delivery to be received	15%	3%	15%	5%	0.980		26%	2%	19%	4%	0.105		0%	0%	1%	0%	0.060	
Share reporting mostly or always received fewer drugs than ordered	35%	4%	38%	7%	0.504		20%	2%	22%	5%	0.620		2%	0%	1%	0%	0.031	*
Share of drugs in stock	64%	1%	66%	2%	0.379		32%	1%	40%	2%	0.000	***	48%	1%	49%	1%	0.063	
Family member or owner works at a public facility													1%	0%	1%	0%	0.826	
Pharmacies registered with the Pharmacy Council													73%	1%	70%	2%	.217	
N	123		55				400		88				1036		608			

Notes: SE: Standard error of the mean. Linked public facilities include all facilities to which a public outpatient visit was linked via the report of the facility by the patient in the household survey. Hospitals include public hospitals and community health centres (CHCs). Primary facilities include primary health centres (PHCs), health and wellness centres (PHCs) and sub-centres (SCs). Distance between the public facility and the private retail pharmacy are based on the GPS coordinates recorded in the survey, with distance estimated based on the geodist command in STATA, with the top 1% of distance trimmed. The denominator for drugs in stock is different for primary facilities (41 drugs) as compared to hospitals and private retail pharmacies (58 drugs). Statistical significance of difference in means represented by p-values: * <.05, ** <.01, ***<.001.

Figure G.1: Outpatient visits among primary facilities

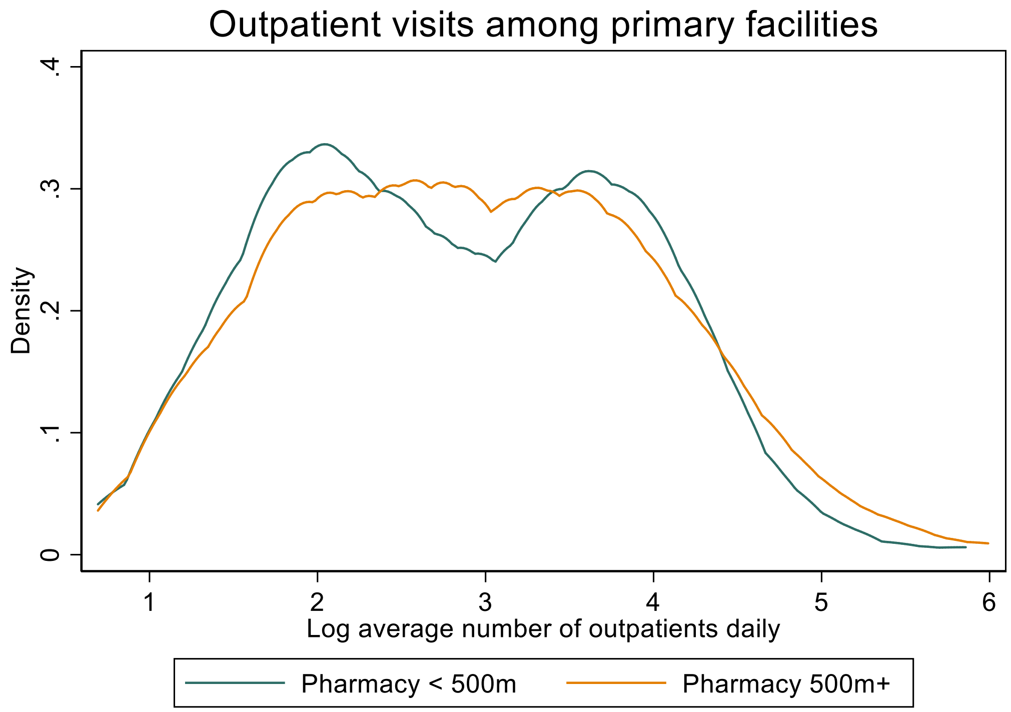

Figure G.2: Out-of-pocket drug costs according to whether patients obtained some private drugs versus obtained only public drugs

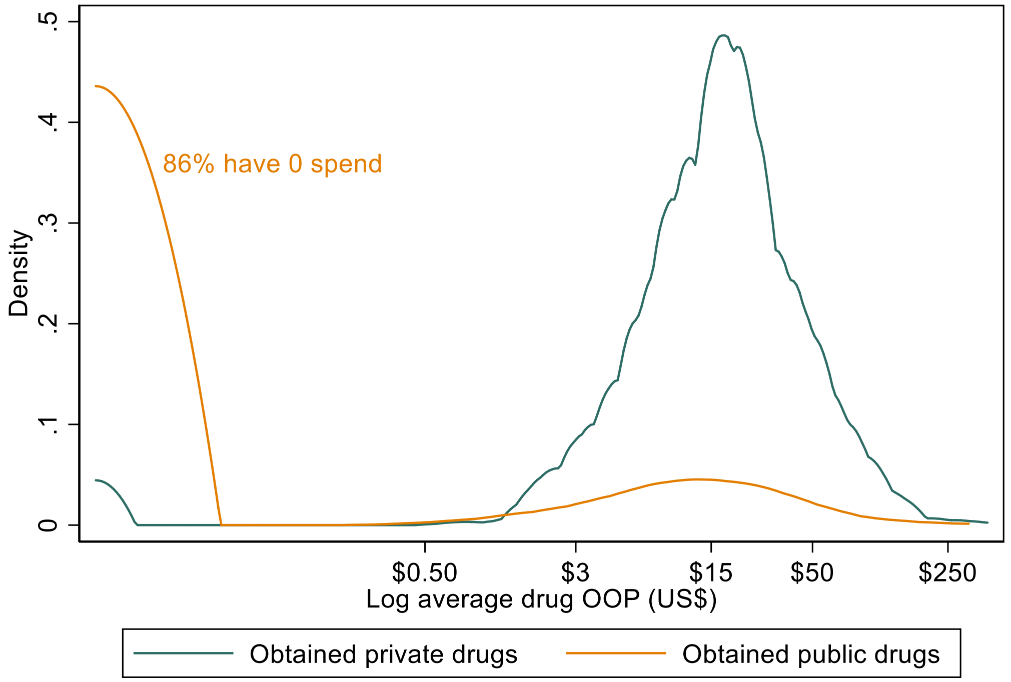

Table G.5 Results from linear probability model of share of drugs in stock by hospitals and primary facilities (Table 3)

	(1)	(2)	(3)	(4)	(5)	(6)
	Hospitals			Primary facilities		
Log distance to nearest pharmacy (kms)	-0.0111	0.00675	0.0138	0.0182*	0.0219*	0.0302**
	(0.686)	(0.827)	(0.659)	(0.036)	(0.013)	(0.002)
Hospitals	-0.0350	0.0125	-0.0131			
	(0.314)	(0.759)	(0.797)			
PHCs				0.264***	0.227***	0.220***
				(0.000)	(0.000)	(0.000)
Whether have inpatient care		0.124*	0.147*			0.0537
		(0.035)	(0.024)			(0.103)
Rural			-0.0813*		-0.00173	0.0404
			(0.030)		(0.979)	(0.583)
Log number of staff			0.00316		0.0380*	0.0371
			(0.890)		(0.043)	(0.068)
Severe water shortages			-0.0126		-0.00754	-0.0125
			(0.700)		(0.621)	(0.447)
Electronic ordering of drugs			0.0194			-0.0783*
			(0.472)			(0.015)
Computerized tracking of drug stocks			0.0241			0.0909*
			(0.429)			(0.048)
Whether takes 2 or more weeks to receive drugs			-0.0179			-0.0601***
			(0.608)			(0.001)
Nearest pharmacy is Jan Aushadhi			0.0176			-0.0278
			(0.683)			(0.463)
Constant	0.651***	0.527***	0.543***	0.210***	0.199**	0.173*
	(0.000)	(0.000)	(0.000)	(0.000)	(0.002)	(0.014)
N	117	117	116	395	388	332

Notes: CHC: Community Health Centre; PHC: Primary Health Centre; SC: Sub-Centre; HWC: Health and Wellness Centre. Standard errors clustered by facility. P-values in parentheses: * p<0.05, ** p<0.01, *** p<0.001.

Table G.6 Results from linear probability model of private drugs and catastrophic health expenditure (CHE) in hospitals (Table 1)

	(1)	(2)	(3)	(4)	(5)	(6)
	Private drugs obtained			CHE due to drugs		
Log non-health expenditure per year (USD)	0.00548	0.00203	0.0122	-0.152***	-0.146***	-0.136***
	(0.884)	(0.959)	(0.741)	(0.000)	(0.000)	(0.000)
Illiterate	-0.130*	-0.145*	-0.149**	0.0223	0.0215	0.0205
	(0.018)	(0.012)	(0.006)	(0.654)	(0.708)	(0.724)
Share of drugs in stock	-0.452*	-0.481*	-0.357	-0.373*	-0.327	-0.396
	(0.027)	(0.047)	(0.152)	(0.033)	(0.118)	(0.103)
Log distance to nearest pharmacy (km)	-1.896*	-1.598	-1.628*	0.0994	0.108	0.111
	(0.025)	(0.079)	(0.032)	(0.466)	(0.354)	(0.335)
CHC	0.0284	0.0330	0.104	-0.0804	-0.0646	-0.131
	(0.643)	(0.622)	(0.331)	(0.070)	(0.152)	(0.067)
Whether provide inpatient care		0.119	-0.0205		-0.0115	0.108
		(0.538)	(0.924)		(0.918)	(0.539)
Female		0.0375	0.0589		0.0300	0.0319
		(0.526)	(0.277)		(0.357)	(0.336)
Poor/Fair self-rated health		-0.0537	-0.0576		0.0596	0.0688
		(0.321)	(0.251)		(0.229)	(0.186)
Diagnosis of a Chronic Condition		0.0580	0.0510		-0.0200	-0.0199
		(0.343)	(0.417)		(0.698)	(0.709)
Whether attended closest government facility		-0.0893*	-0.0750		-0.0505	-0.0483
		(0.034)	(0.078)		(0.186)	(0.220)
Used care for fever		0.0280	0.0519		-0.0201	-0.0154

		(0.658)	(0.408)		(0.604)	(0.695)
Used care for childbirth-related services		-0.244	-0.259		-0.109	-0.0989
		(0.225)	(0.163)		(0.168)	(0.213)
Rural residence			0.0839			0.0227
			(0.335)			(0.559)
Log number of staff present			0.0426			-0.0329
			(0.271)			(0.374)
Severe water shortage			-0.0377			-0.0176
			(0.606)			(0.654)
Electronic ordering of drugs			0.0407			0.00879
			(0.503)			(0.857)
Computerized tracking of drug stocks			0.0958			-0.0170
			(0.208)			(0.698)
Takes 2 weeks or more to receive drugs			0.0156			-0.00239
			(0.881)			(0.955)
Closest pharmacy is a Jan Aushadhi pharmacy			-2.286***			-0.207
			(0.000)			(0.054)
Constant	1.078***	1.031**	0.664*	1.512***	1.472***	1.464***
	(0.001)	(0.003)	(0.039)	(0.000)	(0.000)	(0.000)
N	569	567	567	598	596	596

Notes: CHC: Community Health Centre; PHC: Primary Health Centre; SC: Sub-Centre; HWC: Health and Wellness Centre. Standard errors clustered by facility. P-values in parentheses: * p<0.05, ** p<0.01, *** p<0.001.

Table G.7 Results from linear probability model of number of drugs and pharmacy referrals in hospitals (Table 1)

	(7)	(8)	(9)	(10)	(11)	(12)
	Number of drugs obtained			Referred to a pharmacy		
Log non-health expenditure per year (USD)	0.0244	0.0614	0.0394	0.0128	0.0119	0.00318

	(0.895)	(0.731)	(0.816)	(0.583)	(0.660)	(0.892)
Illiterate	0.206	0.212	0.241	0.0442	0.0168	0.0248
	(0.393)	(0.403)	(0.359)	(0.378)	(0.753)	(0.637)
Share of drugs in stock	-1.223	-0.950	-0.716	-0.349	-0.313	-0.0751
	(0.176)	(0.366)	(0.502)	(0.163)	(0.270)	(0.761)
Log distance to nearest pharmacy (km)	-2.607**	-2.583***	-2.496***	-0.855	-0.717	0.249
	(0.003)	(0.000)	(0.000)	(0.081)	(0.158)	(0.582)
CHC	-0.00360	0.113	0.321	-0.0732	-0.0482	0.120
	(0.989)	(0.650)	(0.318)	(0.200)	(0.426)	(0.117)
Whether provide inpatient care		-0.0328	-0.311		-0.115	-0.443*
		(0.957)	(0.684)		(0.486)	(0.013)
Female		0.274	0.248		0.0311	0.0359
		(0.183)	(0.202)		(0.405)	(0.372)
Poor/Fair self-rated health		0.228	0.195		0.00272	0.0116
		(0.303)	(0.371)		(0.965)	(0.841)
Diagnosis of a Chronic Condition		-0.125	-0.119		0.121	0.0986
		(0.649)	(0.680)		(0.097)	(0.150)
Whether attended closest government facility		-0.508	-0.520*		-0.0256	-0.0478
		(0.056)	(0.039)		(0.688)	(0.413)
Used care for fever		-0.106	-0.0913		0.0105	0.0181
		(0.571)	(0.653)		(0.803)	(0.667)
Used care for childbirth-related services		-0.673*	-0.697*		-0.0293	-0.0529
		(0.026)	(0.031)		(0.776)	(0.652)
Rural residence			-0.0564			-0.0941
			(0.865)			(0.055)
Log number of staff present			0.0788			0.101***
			(0.617)			(0.000)
Severe water shortage			-0.0983			-0.0555

			(0.772)			(0.383)
Electronic ordering of drugs			0.0689			0.121**
			(0.729)			(0.005)
Computerized tracking of drug stocks			0.134			-0.0106
			(0.514)			(0.753)
Takes 2 weeks or more to receive drugs			-0.185			0.117
			(0.505)			(0.098)
Closest pharmacy is a Jan Aushadhi pharmacy			1.261			-0.145
			(0.056)			(0.270)
Constant	3.824**	3.685**	3.552**	0.373	0.422	0.231
	(0.009)	(0.004)	(0.006)	(0.171)	(0.059)	(0.215)
N	597	595	595	569	567	567

Notes: CHC: Community Health Centre; PHC: Primary Health Centre; SC: Sub-Centre; HWC: Health and Wellness Centre. Standard errors clustered by facility. P-values in parentheses: * p<0.05, ** p<0.01, *** p<0.001.

Table G.8 Results from linear probability model of private drugs and catastrophic health expenditure (CHE) in primary facilities (Table 1)

	(1)	(2)	(3)	(4)	(5)	(6)
	Private drugs			CHE due to drugs		
Log non-health expenditure per year (USD)	-0.0131	-0.00779	0.0252	-0.186***	-0.180***	-0.188***
	(0.809)	(0.882)	(0.624)	(0.000)	(0.000)	(0.000)
Illiterate	0.0352	0.0364	0.0328	0.0917	0.0884	0.0193
	(0.641)	(0.642)	(0.697)	(0.271)	(0.284)	(0.799)
Share of drugs in stock	0.233	0.330	0.378	0.199	0.176	0.105
	(0.141)	(0.097)	(0.156)	(0.072)	(0.174)	(0.534)
Log distance to nearest pharmacy (km)	-0.104*	-0.109*	-0.0181	-0.0656**	-0.0893***	-0.0885**

	(0.019)	(0.041)	(0.705)	(0.003)	(0.000)	(0.006)
SC/HWC	0.00714	0.0264	0.0784	0.0794	0.0245	0.0243
	(0.943)	(0.836)	(0.604)	(0.265)	(0.795)	(0.793)
Rural residence		0.396	0.506*		0.0383	0.0841
		(0.081)	(0.022)		(0.621)	(0.254)
Whether provide inpatient care		-0.0230	0.0406		0.146**	0.158**
		(0.839)	(0.648)		(0.004)	(0.008)
Log number of staff present		-0.0184	0.0718		-0.0577	-0.0615
		(0.792)	(0.281)		(0.125)	(0.218)
Severe water shortage		-0.0667	-0.0895		0.0672	0.0524
		(0.489)	(0.427)		(0.358)	(0.566)
Female			0.0237			0.0352
			(0.733)			(0.398)
Poor/Fair self-rated health			0.0710			0.0187
			(0.433)			(0.777)
Diagnosis of a Chronic Condition			0.0325			0.0838
			(0.709)			(0.427)
Attended closest government facility			-0.155			-0.0292
			(0.142)			(0.708)
Used care for fever			-0.0608			0.0548
			(0.507)			(0.291)
Used care for childbirth-related services			0.266			-0.223*
			(0.170)			(0.022)
Electronic ordering of drugs			0.0570			-0.0404
			(0.621)			(0.471)
Computerized tracking of drug stocks			-0.535***			0.0491
			(0.001)			(0.641)
Takes 2 weeks or more to receive drugs			-0.0680			-0.0719
			(0.409)			(0.141)

Closest pharmacy is a Jan Aushadhi pharmacy			-0.316*			-0.368**
			(0.011)			(0.005)
Constant	0.814*	0.385	0.0205	1.350***	1.341***	1.391***
	(0.030)	(0.450)	(0.972)	(0.000)	(0.000)	(0.000)
N	234	228	208	245	239	219

Notes: CHC: Community Health Centre; PHC: Primary Health Centre; SC: Sub-Centre; HWC: Health and Wellness Centre. Standard errors clustered by facility. P-values in parentheses: * p<0.05, ** p<0.01, *** p<0.001.

Table G.9 Results from linear probability model of number of drugs and pharmacy referrals in primary facilities (Table 1)

	(7)	(8)	(9)	(10)	(11)	(12)
	Number of drugs			Pharmacy referral		
Log non-health expenditure per year (USD)	-0.0454	0.0415	0.0657	-0.0144	-0.0142	0.0118
	(0.776)	(0.800)	(0.679)	(0.725)	(0.735)	(0.807)
Illiterate	-0.169	-0.179	-0.160	-0.0163	-0.0315	-0.0126
	(0.427)	(0.390)	(0.537)	(0.813)	(0.624)	(0.826)
Share of drugs in stock	1.232	1.055	0.996	-0.381*	-0.508*	-0.257
	(0.142)	(0.189)	(0.350)	(0.030)	(0.017)	(0.232)
Log distance to nearest pharmacy (km)	-0.0703	-0.0364	-0.0100	-0.0356	0.00879	0.00251
	(0.543)	(0.754)	(0.942)	(0.273)	(0.813)	(0.960)
SC/HWC	0.357	0.103	0.0750	-0.0362	-0.0673	0.105
	(0.311)	(0.796)	(0.871)	(0.732)	(0.638)	(0.479)
Rural residence		1.295**	1.219*		0.0636	0.151
		(0.008)	(0.029)		(0.452)	(0.258)
Whether provide inpatient care		-0.100	-0.187		-0.169**	-0.152
		(0.625)	(0.430)		(0.004)	(0.068)
Log number of staff present		-0.0658	-0.129		0.0451	0.110

		(0.634)	(0.511)		(0.410)	(0.110)
Severe water shortage		0.602*	0.512		0.115	0.0949
		(0.041)	(0.099)		(0.312)	(0.429)
Female			0.0854			0.0486
			(0.629)			(0.420)
Poor/Fair self-rated health			-0.485			-0.103
			(0.087)			(0.183)
Diagnosis of a Chronic Condition			0.665*			0.107
			(0.046)			(0.132)
Attended closest government facility			-0.386			-0.0396
			(0.212)			(0.714)
Used care for fever			-0.0595			-0.149
			(0.771)			(0.170)
Used care for childbirth-related services			0.442			-0.440**
			(0.189)			(0.003)
Electronic ordering of drugs			0.0520			-0.250***
			(0.846)			(0.000)
Computerized tracking of drug stocks			0.0450			-0.0172
			(0.929)			(0.898)
Takes 2 weeks or more to receive drugs			0.231			-0.0383
			(0.498)			(0.735)
Closest pharmacy is a Jan Aushadhi pharmacy			-2.126***			-0.488**
			(0.000)			(0.007)
Constant	2.766*	0.995	1.245	0.477	0.407	0.0772
	(0.038)	(0.507)	(0.431)	(0.153)	(0.260)	(0.863)
N	245	239	219	234	228	208

Notes: CHC: Community Health Centre; PHC: Primary Health Centre; SC: Sub-Centre; HWC: Health and Wellness Centre. Standard errors clustered by facility. P-values in parentheses: * p<0.05, ** p<0.01, *** p<0.001.

Table G.10 Sensitivity analysis of outcomes included in Table 1

Outcome	Model	Hospitals					Primary facilities						
		Share of drugs in stock at facility used	P-value	Log distance to nearest pharmacy (kms)	P-value	N	Share of drugs in stock at facility used	P-value	Log distance to nearest pharmacy (kms)	P-value	N	Sample difference covariates	Pharmacy management covariates
CHE due to drugs	OLS	**-0.373***	0.033	0.0994	0.466	598	0.199	0.072	**-0.0656****	0.003	245		
		-0.327	0.118	0.108	0.354	596	0.197	0.118	**-0.0784*****	0	237	X	
		-0.396	0.103	0.111	0.335	596	0.105	0.534	**-0.0885****	0.006	219	X	X
	Logit	**-3.502***	0.02	0.879	0.457	598	2.036	0.086	**-0.808****	0.01	245		
		**-3.828***	0.041	1.104	0.125	596	2.040	0.112	**-1.204****	0.007	228	X	
		**-4.677***	0.012	1.274	0.053	594	0.621	0.73	**-0.996***	0.039	203	X	X
	GEE-Binomial	-2.152	0.099	0.930	0.253	598	1.610	0.189	**-0.869*****	0.001	245		
		-2.660	0.093	0.896	0.271	596	1.247	0.31	**-1.089****	0.001	228	X	
		-2.865	0.09	1.021	0.229	594						X	X
	GLM-Binomial collapsed by facility	-1.199	0.329	0.898	0.28	53	1.132	0.395	**-0.673***	0.018	80		
		-0.910	0.685	0.679	0.437	52	0.910	0.516	**-0.859***	0.014	78	X	
		-0.802	0.747	1.166	0.222	52	1.826	0.349	**-0.973***	0.015	71	X	X
Number of Drugs	OLS	-1.223	0.176	**-2.607****	0.003	597	1.232	0.142	-0.0703	0.543	245		
		-0.950	0.366	**-2.583*****	0	595	1.055	0.189	-0.0364	0.754	239	X	
		-0.716	0.502	**-2.496*****	0	595	0.996	0.35	-0.0100	0.942	219	X	X
	GEE-Gaussian	-1.032	0.233	**-1.992****	0.003	597	**1.307***	0.024	-0.00463	0.968	245		
		-0.578	0.555	**-2.095****	0.002	595	**1.291***	0.02	-0.0217	0.842	239	X	
							**1.096***	0.05	-0.0533	0.598	219	X	X
	Collapsed by facility	-0.630	0.439	**-2.042*****	0	53	**1.909***	0.022	-0.0585	0.717	80		
		-0.0789	0.953	**-2.258*****	0	52	**2.290****	0.008	-0.0883	0.583	78	X	
		0.430	0.783	**-2.371*****	0	52	**2.252***	0.028	-0.0872	0.642	71	X	X
	OLS	-0.349	0.163	-0.855	0.081	569	**-0.381***	0.03	-0.0356	0.273	234		

Referral to a particular pharmacy		-0.313	0.27	-0.717	0.158	567	**-0.508***	0.017	0.00879	0.813	228	X	
		-0.0751	0.761	0.249	0.582	567	-0.257	0.232	0.00251	0.96	208	X	X
	Logit	-2.734	0.132	-7.885	0.105	569	**-3.253***	0.024	-0.300	0.337	234		
		-2.434	0.241	-7.518	0.184	567	**-4.415***	0.01	0.0684	0.832	190	X	
		0.123	0.961	0.792	0.877	565	-2.067	0.274	0.0302	0.946	145	X	X
	GEE-Binomial	**-3.143***	0.027	-6.066	0.104	569	-2.011	0.161	-0.0942	0.736	234		
		-3.073	0.054	-6.320	0.118	567	-2.643	0.074	0.0338	0.909	190	X	
		-1.554	0.425	-2.371	0.602	565	-2.168	0.212	-0.0424	0.89	145	X	X
	GLM-Binomial collapsed by facility	**-3.281****	0.008	-5.655	0.093	53	-2.293	0.06	-0.170	0.487	80		
		**-4.616***	0.021	-6.151	0.126	52	**-2.697***	0.034	-0.0214	0.936	78	X	
		-0.0920	0.973	-3.715	0.44	52	-2.948	0.125	-0.0541	0.864	71	X	X
Private drugs obtained	OLS	**-0.452***	0.027	**-1.896***	0.025	569	0.233	0.141	**-0.104***	0.019	234		
		**-0.481***	0.047	-1.598	0.079	567	0.330	0.097	**-0.109***	0.041	228	X	
		-0.357	0.152	**-1.628***	0.032	567	0.378	0.156	-0.0181	0.705	208	X	X
	Logit	**-2.459***	0.026	**-9.329***	0.022	569	1.518	0.157	**-0.582***	0.015	234		
		-2.692	0.053	-7.998	0.066	567	2.094	0.101	**-0.611***	0.024	228	X	
		-2.472	0.112	**-8.382***	0.025	565	2.562	0.112	-0.137	0.597	205	X	X
	GEE-Binomial	**-2.634***	0.037	**-7.398****	0.005	569	-0.215	0.849	**-0.419***	0.045	234		
		**-3.001***	0.036	**-6.705***	0.014	567	0.420	0.725	-0.366	0.1	228	X	
		-2.441	0.083	-4.558	0.114	565	0.726	0.625	-0.182	0.478	205	X	X
	GLM-Binomial	**-2.346***	0.012	**-8.169*****	0	53	-0.625	0.477	-0.208	0.2	80		
		**-3.227***	0.038	**-5.727***	0.018	52	-0.419	0.655	-0.158	0.369	78	X	
		-0.401	0.828	-5.181	0.064	52	-0.347	0.768	0.0287	0.887	71	X	X

Notes: CHE: catastrophic health expenditure measured at the 10% of consumption expenditure threshold level; OLS: ordinary least squares; GEE: generalized estimating equation; GLM: generalized linear model. OLS, Logit and GEE were clustered by facility. All models include log non-health expenditure, whether the respondent was literate and fixed effects on level of facility. Sample difference covariates included all indicators that were statistically significantly different in the all versus linked samples, which were: gender, rurality, poor/fair self-rated health, diagnosis with a chronic condition, closest government facility used, use of care for fever or child birth, whether the facility had an inpatient department, the natural log of the number of staff present on the day of the survey and whether the facility faces severe water shortages. Pharmacy management covariates were: electronic ordering of drugs, computerized tracking of drugs, receipt of drugs often takes more than two weeks and whether the closest pharmacy was a Jan Aushadhi pharmacy. 8 primary facilities did not have the pharmacy management covariates recorded.

Table G.11: Regression of patient rating of excellent or good needs met on private drugs obtained and any drugs obtained (Table 2)

	(1)	(2)	(3)	(4)	(5)	(6)
		Hospitals			Primary facilities	
Private drugs obtained	-0.0231	-0.0202	-0.0391	-0.124*	-0.132*	-0.190*
	(0.665)	(0.613)	(0.445)	(0.021)	(0.018)	(0.029)
Any drugs obtained	0.273**	0.218**	0.268**	-0.0168	-0.00143	0.129
	(0.006)	(0.004)	(0.008)	(0.773)	(0.980)	(0.255)
Log OOP costs (USD)	0.00423	0.0151	0.00958	0.00889	0.0129	0.0424
	(0.841)	(0.291)	(0.649)	(0.655)	(0.553)	(0.139)
Any test obtained	-0.0645	-0.0117	-0.0371	0.0185	0.0282	-0.0612
	(0.327)	(0.797)	(0.581)	(0.680)	(0.547)	(0.476)
Hospital			-0.0204			
			(0.688)			
CHC	0.0256	0.0339				
	(0.552)	(0.393)				
SC/HWC				0.0170	0.0137	-0.0256
				(0.732)	(0.801)	(0.771)
Log non-health expenditure per year (USD)	-0.0463	-0.0323	-0.0547*	-0.0124	-0.0293	-0.00996
	(0.086)	(0.163)	(0.041)	(0.661)	(0.342)	(0.834)
Illiterate	0.0205	0.0369	0.0305	-0.0219	0.00364	0.0462
	(0.717)	(0.426)	(0.615)	(0.663)	(0.948)	(0.553)
Poor/Fair self-rated health		-0.0893	-0.101		-0.0414	-0.104
		(0.074)	(0.180)		(0.430)	(0.149)
Female		-0.00771	-0.0277		-0.0277	-0.0566
		(0.803)	(0.474)		(0.510)	(0.381)
Rural residence		-0.0663	-0.0175		-0.0567	-0.0863
		(0.121)	(0.740)		(0.437)	(0.363)
Attended closest government facility		-0.0409	-0.0336		-0.0139	-0.113*
		(0.305)	(0.547)		(0.831)	(0.044)
Used care for fever		-0.0297	-0.0254		-0.0291	0.0468
		(0.447)	(0.653)		(0.557)	(0.402)
Used care for childbirth		-0.0681	-0.269		0.165***	0.103
		(0.484)	(0.059)		(0.000)	(0.421)
Diagnosed with chronic condition		0.0160	0.00313		-0.0306	0.171*
		(0.667)	(0.951)		(0.572)	(0.035)
Scheduled tribe		-0.0360	-0.0695		-0.0172	-0.0138
		(0.555)	(0.327)		(0.703)	(0.804)
Scheduled caste		0.0735	0.0378		-0.113	-0.285*
		(0.068)	(0.470)		(0.177)	(0.017)
Serve inpatient			0.0371			-0.0980

			(0.695)			(0.091)
Log staff present			0.00175			-0.0753
			(0.954)			(0.157)
Severe water shortage			0.0542			-0.155
			(0.340)			(0.111)
Constant	0.882***	0.911***	1.008***	1.011***	1.237***	1.177**
	(0.000)	(0.000)	(0.000)	(0.000)	(0.000)	(0.003)
N	552	898	552	430	430	219

Notes: CHC: Community Health Centre; PHC: Primary Health Centre; SC: Sub-Centre; HWC: Health and Wellness Centre. Standard errors clustered by facility and primary sampling unit. P-values in parentheses: * p<0.05, ** p<0.01, *** p<0.001.

Table G.12: Regression of patient rating of excellent or good quality on private drugs obtained and any drugs obtained (Table 2)

	(7)	(8)	(9)	(10)	(11)	(12)
	Hospitals			Primary facilities		
Private drugs obtained	0.0151	0.0112	-0.0222	-0.0334	-0.0662	-0.0662
	(0.757)	(0.819)	(0.702)	(0.589)	(0.287)	(0.540)
Any drugs obtained	0.189*	0.184*	0.288**	0.0162	0.0306	0.112
	(0.025)	(0.027)	(0.004)	(0.881)	(0.759)	(0.453)
Log OOP costs (USD)	0.0241	0.0292	0.0169	-0.0503*	-0.0378	-0.00892
	(0.122)	(0.081)	(0.467)	(0.048)	(0.130)	(0.820)
Any test obtained	-0.0410	-0.0369	-0.0683	0.0902	0.0781	-0.0127
	(0.430)	(0.471)	(0.357)	(0.179)	(0.238)	(0.874)
Hospital	-0.0757*		-0.106			
	(0.046)		(0.052)			
CHC		0.0901*				
		(0.026)				
SC/HWC				0.0285	0.00681	0.0473
				(0.680)	(0.921)	(0.685)
Log non-health expenditure per year (USD)	0.00946	-0.00106	-0.0376	-0.00185	0.00451	-0.0130
	(0.717)	(0.969)	(0.243)	(0.966)	(0.908)	(0.795)
Illiterate	0.0432	0.0600	0.0420	-0.0394	-0.0176	-0.0691
	(0.356)	(0.237)	(0.515)	(0.505)	(0.797)	(0.489)
Poor/Fair self-rated health		0.0155	-0.0383		0.0515	-0.0748
		(0.748)	(0.543)		(0.403)	(0.419)
Female		-0.0345	-0.0360		-0.0100	-0.00360
		(0.306)	(0.390)		(0.879)	(0.970)

Rural residence		-0.110**	-0.0303		0.149	-0.133
		(0.008)	(0.581)		(0.193)	(0.241)
Attended closest government facility		-0.00727	-0.00386		0.171*	0.0767
		(0.864)	(0.942)		(0.045)	(0.446)
Used care for fever		-0.0190	-0.00974		0.0491	0.150
		(0.648)	(0.862)		(0.439)	(0.063)
Used care for childbirth		-0.0102	-0.177		0.255***	0.106
		(0.914)	(0.183)		(0.001)	(0.537)
Diagnosed with chronic condition		-0.0522	-0.0290		0.0819	0.318***
		(0.203)	(0.568)		(0.240)	(0.000)
Scheduled tribe		-0.0365	-0.0806		-0.0289	-0.111
		(0.567)	(0.333)		(0.645)	(0.237)
Scheduled caste		0.0324	-0.0285		-0.0792	-0.292*
		(0.501)	(0.651)		(0.357)	(0.022)
Serve inpatient			-0.149			-0.279**
			(0.085)			(0.009)
Log staff present			0.0309			0.0194
			(0.286)			(0.763)
Severe water shortage			0.0409			-0.142
			(0.542)			(0.144)
Constant	0.477*	0.589*	0.922**	0.848**	0.469	0.832
	(0.034)	(0.012)	(0.001)	(0.003)	(0.129)	(0.056)
N	900	898	552	430	430	219

Notes: CHC: Community Health Centre; PHC: Primary Health Centre; SC: Sub-Centre; HWC: Health and Wellness Centre. Standard errors clustered by facility and primary sampling unit. P-values in parentheses: * p<0.05, ** p<0.01, *** p<0.001.

Table G.13: Sensitivity analysis of patient ratings among primary facilities, stratified by distance to the nearest pharmacy

	(1)	(2)	(3)	(4)	(5)	(6)	(7)	(8)	(9)	(10)	(11)	(12)
	Patient rating of excellent/good needs						Patient rating of excellent/good quality					
	Pharmacy <500 meters	Pharmacy 500+ meters	Pharmacy <500 meters	Pharmacy 500+ meters	Pharmacy <500 meters	Pharmacy 500+ meters	Pharmacy <500 meters	Pharmacy 500+ meters	Pharmacy <500 meters	Pharmacy 500+ meters	Pharmacy <500 meters	Pharmacy 500+ meters
Private drugs obtained	-0.244*	-0.170	-0.153	-0.136	-0.152	-0.117	-0.0412	-0.180	-0.0366	-0.159	-0.0307	-0.118
	(0.036)	(0.213)	(0.187)	(0.309)	(0.164)	(0.414)	(0.775)	(0.144)	(0.795)	(0.256)	(0.824)	(0.433)
Any drugs obtained	0.0108	0.155	0.0385	0.172	0.0383	0.212	-0.113	0.0920	-0.0348	0.139	-0.0368	0.250
	(0.941)	(0.491)	(0.791)	(0.367)	(0.768)	(0.315)	(0.597)	(0.673)	(0.868)	(0.508)	(0.859)	(0.268)
Log OOP costs (USD)	0.0321	0.0633	0.0267	0.0584	0.0538	0.0508	-0.0207	0.0179	-0.00861	0.0203	0.0146	0.00621
	(0.480)	(0.176)	(0.479)	(0.125)	(0.134)	(0.242)	(0.669)	(0.688)	(0.872)	(0.696)	(0.785)	(0.906)
Any test obtained	0.0868	-0.142	0.0941	-0.160	0.0429	-0.176	0.118	-0.0863	0.103	-0.110	0.0742	-0.130
	(0.415)	(0.193)	(0.467)	(0.081)	(0.760)	(0.089)	(0.227)	(0.606)	(0.247)	(0.488)	(0.435)	(0.314)
SC/HWC	0.0358	-0.000521	0.0677	-0.0321	0.180	-0.118	-0.0386	0.0928	-0.0564	0.0465	0.184	-0.00174
	(0.760)	(0.994)	(0.592)	(0.712)	(0.301)	(0.206)	(0.733)	(0.522)	(0.622)	(0.737)	(0.218)	(0.991)
Log non-health expenditure per year (USD)	0.0528	-0.0455	0.0683	-0.108	0.0308	-0.0648	0.0962	-0.0245	0.0592	-0.100	0.0299	-0.0664
	(0.307)	(0.482)	(0.278)	(0.110)	(0.630)	(0.337)	(0.129)	(0.763)	(0.370)	(0.227)	(0.651)	(0.435)
Illiterate	0.0419	0.0121	0.0650	0.0471	0.0757	0.0448	-0.195	0.179	-0.224	0.222*	-0.228	0.214*
	(0.685)	(0.907)	(0.584)	(0.554)	(0.514)	(0.556)	(0.150)	(0.067)	(0.131)	(0.013)	(0.109)	(0.038)
Poor/Fair self-rated health			-0.0985	-0.0960	-0.0792	-0.135			-0.0716	0.0495	-0.0639	-0.0107

			(0.302)	(0.476)	(0.395)	(0.385)			(0.530)	(0.684)	(0.597)	(0.940)
Female			-0.0392	-0.144	-0.0609	-0.0898			0.0453	-0.163	0.0386	-0.126
			(0.637)	(0.112)	(0.526)	(0.397)			(0.681)	(0.256)	(0.741)	(0.361)
Rural residence			-0.128	-0.273*	0.00197	-0.230			-0.178	-0.217	-0.138	-0.103
			(0.304)	(0.022)	(0.993)	(0.147)			(0.337)	(0.135)	(0.594)	(0.399)
Attended closest government facility			-0.211*	-0.0379	-0.190	-0.0243			0.123	-0.0386	0.0926	-0.0681
			(0.041)	(0.769)	(0.067)	(0.871)			(0.279)	(0.831)	(0.415)	(0.724)
Used care for fever			-0.0547	0.0907	0.0517	0.0679			0.0492	0.211	0.103	0.187
			(0.517)	(0.245)	(0.568)	(0.356)			(0.615)	(0.057)	(0.232)	(0.078)
Used care for childbirth			0.268*	0.138	0.366*	0.110			0.192	0.215	0.259	0.0921
			(0.013)	(0.483)	(0.022)	(0.604)			(0.295)	(0.423)	(0.220)	(0.757)
Diagnosed with chronic condition			0.230*	0.0275	0.187	0.0703			0.317***	0.224	0.296***	0.294
			(0.046)	(0.856)	(0.071)	(0.690)			(0.000)	(0.189)	(0.001)	(0.060)
Scheduled tribe			0.0461	0.0190	-0.0375	0.0212			-0.120	-0.0529	-0.187	-0.108
			(0.645)	(0.806)	(0.694)	(0.806)			(0.424)	(0.588)	(0.246)	(0.325)
Scheduled caste			-0.283*	-0.175	-0.295**	-0.285			-0.318	-0.0728	-0.332	-0.162
			(0.043)	(0.373)	(0.010)	(0.195)			(0.090)	(0.567)	(0.057)	(0.342)
Serve inpatient					-0.167	-0.0921					-0.134	-0.384*
					(0.146)	(0.339)					(0.504)	(0.037)
Log staff present					-0.0244	-0.0587					0.109	0.0484

					(0.813)	(0.421)					(0.231)	(0.600)
Severe water shortage					-0.358**	0.0509					-0.321*	-0.0211
					(0.003)	(0.623)					(0.020)	(0.856)
Constant	0.488	1.072	0.660	1.827**	0.804	1.517*	0.245	0.894	0.472	1.534*	0.505	1.202
	(0.155)	(0.059)	(0.145)	(0.003)	(0.116)	(0.025)	(0.592)	(0.157)	(0.372)	(0.021)	(0.394)	(0.088)
N	125	104	125	104	124	95	125	104	125	104	124	95

Notes: CHC: Community Health Centre; PHC: Primary Health Centre; SC: Sub-Centre; HWC: Health and Wellness Centre. Standard errors clustered by facility and primary sampling unit. P-values in parentheses: * p<0.05, ** p<0.01, *** p<0.001.

Table G.14: Sensitivity analysis of outcomes included in Table 2

Outcome	Model	Hospitals					Primary facilities					Sample differ-rence covar-iates	Facility covariates
		Private drugs obtained		Any drugs obtained		N	Private drugs obtained		Any drugs obtained		N		
Patient rating of excellent/good needs met	OLS	-0.0159	(0.696)	0.217**	(0.005)	900	-0.124*	(0.021)	-0.0168	(0.773)	430		
		-0.0202	(0.613)	0.218**	(0.004)	898	-0.132*	(0.018)	-0.00143	(0.980)	430	X	
		-0.0391	(0.445)	0.268**	(0.008)	552	-0.190*	(0.029)	0.129	(0.255)	219	X	X
	Logit	-0.105	(0.709)	1.148**	(0.003)	900	-1.039*	(0.035)	-0.318	(0.696)	430		
		-0.144	(0.615)	1.184**	(0.002)	898	-1.115*	(0.024)	-0.193	(0.812)	421	X	
		-0.253	(0.483)	1.436**	(0.005)	552	-1.891*	(0.015)	1.414	(0.155)	206	X	X
	GEE-Binomial	-0.268	(0.415)	1.192**	(0.001)	552	-1.839**	(0.007)	0.887	(0.305)	206	X	X
	GLM-Binomial collapsed by facility	-0.541	(0.600)	1.209	(0.436)	51	-1.694	(0.182)	0.795	(0.606)	72	X	X
Patient rating of excellent/good quality	OLS	0.0151	(0.757)	0.189*	(0.025)	900	-0.0334	(0.589)	0.0162	(0.881)	430		
		0.0112	(0.819)	0.184*	(0.027)	898	-0.0662	(0.287)	0.0306	(0.759)	430	X	
		-0.0222	(0.702)	0.288**	(0.004)	552	-0.0662	(0.540)	0.112	(0.453)	219	X	X
	Logit	0.0826	(0.750)	0.864*	(0.020)	900	-0.247	(0.521)	0.129	(0.852)	430		
		0.0502	(0.849)	0.856*	(0.020)	898	-0.448	(0.251)	0.170	(0.792)	430	X	
		-0.125	(0.721)	1.375**	(0.003)	552	-0.542	(0.448)	1.001	(0.334)	211	X	X
	GEE-Binomial	-0.0934	(0.750)	1.062**	(0.002)	552	-1.385*	(0.016)	1.087	(0.143)	211	X	X
	GLM-Binomial collapsed by facility	1.136	(0.242)	-0.654	(0.646)	51	-1.683	(0.116)	1.848	(0.162)	72	X	X

Notes: OLS: ordinary least squares; GEE: generalized estimating equation; GLM: generalized linear model. OLS, Logit and GEE were clustered by facility. All models include log non-health expenditure, whether the respondent was literate, log out-of-pocket costs for the visit, whether any test was obtained, and fixed effects on level of facility. Sample difference covariates included all indicators that were statistically significantly different in the all versus linked samples, which were: gender, rurality, poor/fair self-rated health, diagnosis with a chronic condition, closest government facility used, use of care for fever or child birth, scheduled caste and scheduled tribe. Facility covariates included:
whether the facility had an inpatient department, the natural log of the number of staff present on the day of the survey, and whether the facility faces severe water shortages.

References

1. Kolenikov S. 2014. Calibrating survey data using iterative proportional fitting. *The Stata Journal.* Volume 14, Number 1, pp. 22=59.

2. Census of India. 2011. Available at: https://censusindia.gov.in/2011census/population_enumeration.html (Accessed December 2020).

3. Reserve Bank of India.2015. Number and Percentage of Population Below Poverty Line. Available at: https://web.archive.org/web/20170602074949/https://www.rbi.org.in/SCRIPTs/PublicationsView.aspx?id=16603 (Accessed April 2021).
